# Phototriggered Complex Motion by Programmable Construction of Light-Driven Molecular Motors in Liquid Crystal Networks

**DOI:** 10.1021/jacs.2c01060

**Published:** 2022-04-05

**Authors:** Jiaxin Hou, Guiying Long, Wei Zhao, Guofu Zhou, Danqing Liu, Dirk J. Broer, Ben L. Feringa, Jiawen Chen

**Affiliations:** † SCNU-UG International Joint Laboratory of Molecular Science and Displays, National Center for International Research on Green Optoelectronics, South China Normal University, Guangzhou 510006, China; ‡ Stratingh Institute for Chemistry, University of Groningen, Nijenborgh 4, 9747AG Groningen, The Netherlands; § SCNU-TUE Joint lab of Device Integrated Responsive Materials (DIRM), Guangdong Provincial Key Laboratory of Optical Information Materials and Technology & Institute of Electronic Paper Displays, South China Academy of Advanced Optoelectronics, South China Normal University, Guangzhou 510006, China; ∥ Stimuli-responsive Functional Materials and Devices, Department of Chemical Engineering and Chemistry, Eindhoven University of Technology, Den Dolech 2, Eindhoven 5600 MB, The Netherlands

## Abstract

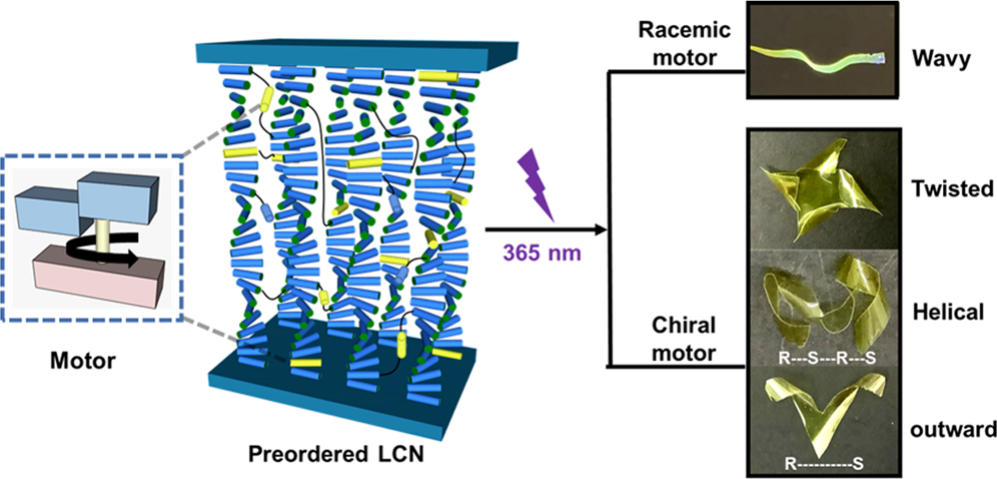

Recent developments in artificial molecular machines have enabled precisely controlled molecular motion, which allows several distinct mechanical operations at the nanoscale. However, harnessing and amplifying molecular motion along multiple length scales to induce macroscopic motion are still major challenges and comprise an important next step toward future actuators and soft robotics. The key to addressing this challenge relies on effective integration of synthetic molecular machines in a hierarchically aligned structure so numerous individual molecular motions can be collected in a cooperative way and amplified to higher length scales and eventually lead to macroscopic motion. Here, we report the complex motion of liquid crystal networks embedded with molecular motors triggered by single-wavelength illumination. By design, both racemic and enantiomerically pure molecular motors are programmably integrated into liquid crystal networks with a defined orientation. The motors have multiple functions acting as cross-linkers, actuators, and chiral dopants inside the network. The collective rotary motion of motors resulted in multiple types of motion of the polymeric film, including bending, wavy motion, fast unidirectional movement on surfaces, and synchronized helical motion with different handedness, paving the way for the future design of responsive materials with enhanced complex functions.

## Introduction

Motion is essentially vital in nature as it supports a broad range of crucial functions for all living systems.^1,2^ It represents a collective action at all length scales.^3,4^ Biological molecular machines transform chemical energy upon external stimuli into specific activities, and the motion at nanoscales is then coordinated and amplified through an ordered approach to mesoscopic and eventually to macroscopic function.^5−7^ The processes are known to have high efficiency, sophistication, and complexity. Typical examples include the fast directional motion of actin filaments driven by myosin, which leads to muscle contraction^8^ and the orthogonally twisted motion with different handedness that afford coiling of plant tendrils.^9^ Inspired by the natural systems, chemists have built artificial molecular machines that utilize chemical, photochemical, electrical, and thermal energy inputs to achieve movement or distinct mechanical operations.^10−19^ These molecular machines are able to perform bending, transitional, and rotary motions, and efforts have been made in the past to integrate the controlled molecular motions to realize specific functions. Chemical synthesizers,^20,21^ molecular shuttle,^22−24^ multitasking catalysts,^25,26^ self-sorting machines,^27,28^ nanocars,^29^ artificial muscle,^30^ transporters,^31,32^ and pumps^33−35^ are illustrative examples of artificial systems with an ever-increasing degree of sophistication at the molecular level. However, harnessing the motion of molecular machines into useful macroscopic function remains a major challenge as motions in solution are confined to the nanoscale and fluids are not endowed with shape or structure capable of mediating mechanical function, and thus, any controlled function at higher length scales is not guaranteed.^36−38^

The key to addressing this challenge is the construction of a hierarchically ordered structure that can sustain the molecular motion cooperatively and eventually lead to successful amplification to a higher scale. One important approach is based on molecular crystals. The stimuli-responsive molecular machines are precisely organized in crystals, and shape change of the molecules induces motion and might lead to the deformation of crystals.^39−43^ Characteristic examples are light-responsive single crystals containing diarylethenes derivatives.^44−47^ Light-responsive crystals have been demonstrated to achieve fast bending and twisting into chiral shapes, but enhanced mechanical function is not realized by this approach due to the limitations of crystals (softness, stability, flexibility, elastic modulus, etc.).^44−47^ Alternatively, another important approach to amplifying molecular motion across length scales relies on liquid crystal materials. Molecular machines can organize in line with liquid crystal molecules, which are sufficiently orientated due to the inherent properties of liquid crystals. The long-range orientational order of liquid crystals promotes amplification of molecular motion of the doped molecular machines from the nanoscale upward. The shape changes of molecular machines induce anisotropic deformation of the liquid crystal materials.^48−51^ Azobenzene derivatives have been widely used for stimuli-responsive liquid crystal materials.^52−56^ Azobenzenes undergo photoisomerization upon light irradiation, changing from a rodlike (*E*) structure to a crescentlike (*Z*) structure. The rodlike structure is compatible with the liquid crystal (LC) order, while the crescentlike structure disrupts it. The disorder created by the photoisomerization of azobenzenes leads to anisotropic deformation of liquid crystal materials, which is further transformed into a large variety of mechanical motions, such as bending,^52^ curling,^57^ twisting,^58^ and winding.^59^

Among the above functions, motility and orthogonal helical motion are two important but largely unexploited functions as the challenge is that amplification of nanoscale motion has to reach beyond one-dimensional transformation, i.e., bending of the substrate. The transformation of shape changes into motility is not always trivial due to the reason that it requires nonsymmetric interactions of the substrates with the surroundings. In pioneering studies reported by White,^60^ Wiersma,^61^ and Broer,^62^ different mechanisms were employed including rolling, inching, and fast oscillation to realize the directional movement of LC materials. In addition, the transmission of chiral motion at the molecular level into helical motion at macroscopic scales, i.e., amplification of chirality, and further conversion into mechanical work provided another fascinating challenge as well. It again requires the delicate design of the system as amplification of molecular motion has to undergo in a preferred handedness. Katsonis and co-worker achieved the helical tendril-like motion using light of distinct wavelengths, a chiral dopant, and an azobenzene photoswitch, taking advantage of the specific morphology and alignment in the LC material.^58^ Furthermore, a system that can perform both types of the above functions, i.e., motility and orthogonal helical motion, has not been realized but is highly desired as it represents a major next step for designing artificial responsive materials with advanced complexity, sophistication, and versatility of mechanical motion.

**Figure 1:**
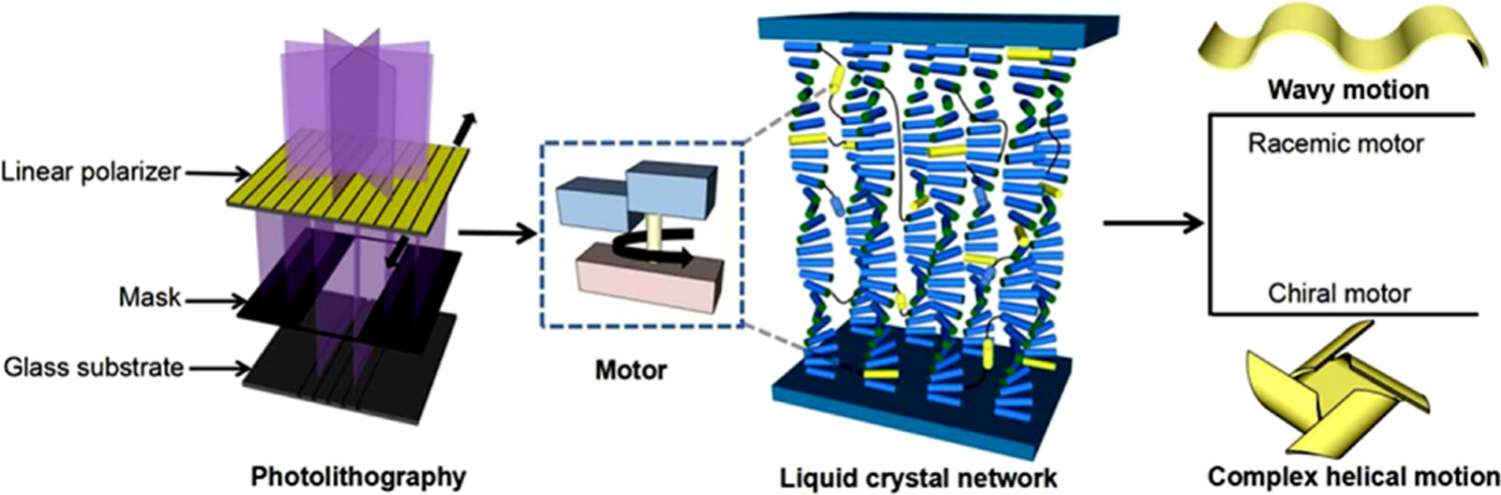
Representation of programmable construction of polymer films, molecular motors in liquid crystal networks by photolithography. Light-driven rotary motors are organized in liquid crystal networks with the designed alignment. The polymeric liquid crystal films containing racemic motors are able to perform fast wavy motion and can move on surfaces, while films with enantiomerically pure motors show complex helical motion upon being exposed to UV-light irradiation.

In the present study, we report a phototriggered liquid crystal network (LCN) that is able to conduct fast directional motility and allows complex orthogonal helical motion with different handedness by the use of rotary molecular motors (Figure 1). Inherently chiral molecular motors based on overcrowded alkenes can rotate unidirectionally driven by light in a noninvasive manner. The rotary cycle of motors involves not only geometrical change of the molecule but also helicity change, which distinguishes motors from most other molecular switches.^10,11,63−65^ Molecular motors have been applied to achieve macroscopic functions,^66^ including changing the wettability of surfaces^67^ and photoactuation of a supramolecular polymer.^30^

In nature, complex shape deformations and functions are often driven by hierarchical structuring of molecular systems, such as the cellular ensembles forming muscles and the tissue of plants. Meanwhile, a liquid crystal network is constructed by liquid crystalline molecules whose orientation can be modulated using alignment layers and fixed by photopolymerization, resulting in hierarchical materials with a defined microstructure over macroscopic length scales.^68^ Therefore, we envisioned that, by taking advantage of the long-range orientational order of liquid crystals, the unidirectional rotation of molecular motors can be effectively amplified and the rotary motion and the accompanied helicity change can be cooperatively transmitted and thus result in complex functions. Our previous studies have shown that the molecular motor is compatible with liquid crystals both noncovalently^69−71^ and covalently^72^ while retaining its rotary motion. We expected that, by programmably embedding molecular motors in LCN, lifelike motion with enhanced complexity can be achieved. In the present study, we employed photolithography, which is a technique used in the precise microfabrication for patterning surfaces of substrates,^73^ to better incorporate motors with defined alignments in LCN. The resulting polymeric films with preordering of racemic or homochiral motors can induce not only fast wavy motion but also synchronized helical motion with different handedness as well as upon light irradiation (Figure 1), enabling complex shape changes and motion on demand, depending on the chirality of the system.

## Results and Discussion

**Figure 2:**
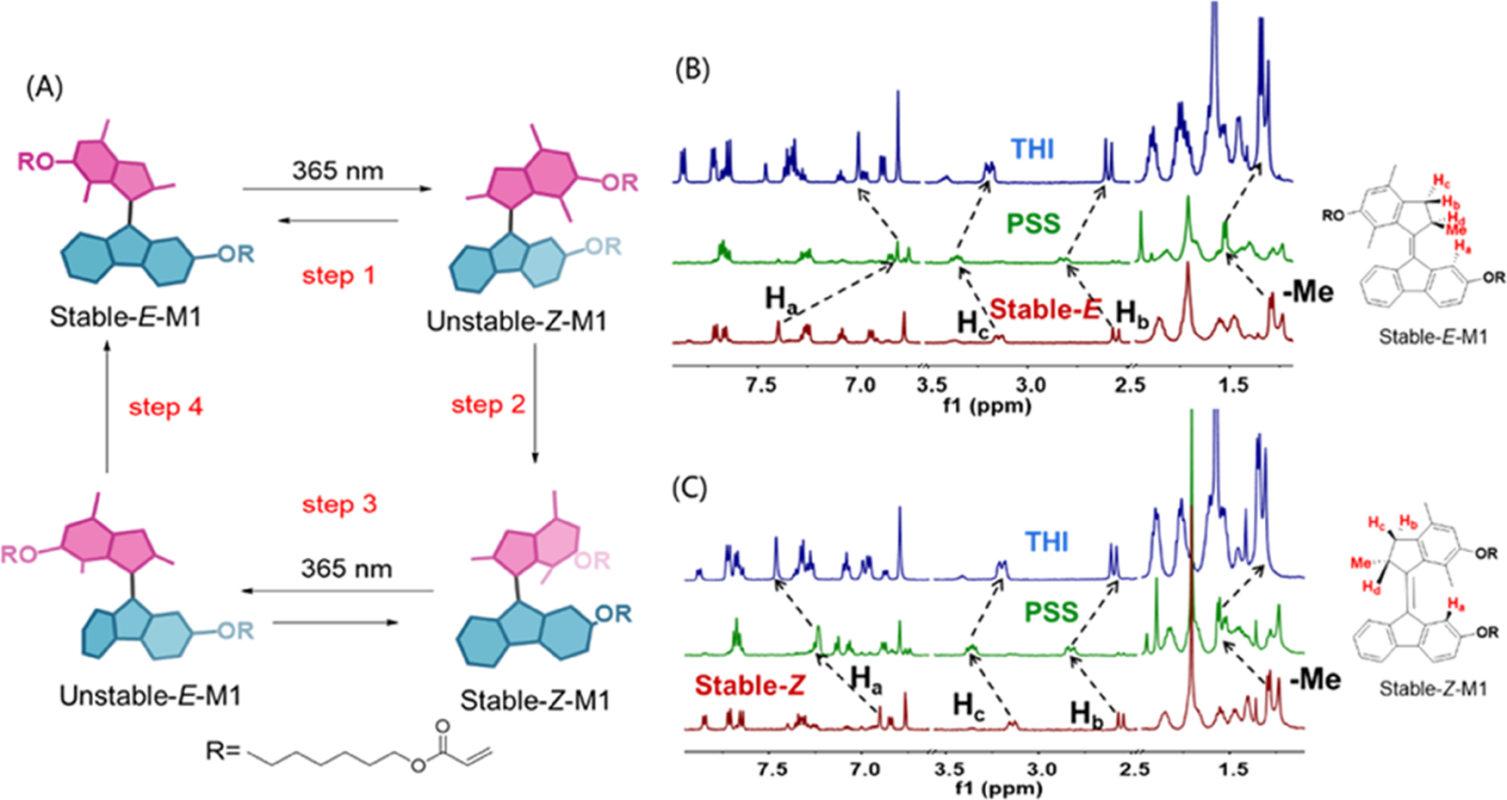
Light-driven rotation of molecular motor **M1**. (A) Rotary cycle of **M1** (only one enantiomer is shown here). Steps 1 and 3: photoisomerization; steps 2 and 4: thermal helix inversion. (B) Partial ^1^H NMR of *E*-**M1** (CD_2_Cl_2_, −40 °C): (red) stable-*E*-**M1**, before irradiation (λ ≥ 365 nm); (green) after irradiation; and (blue) after standing at room temperature in the dark for 2 h. (C) Partial ^1^H NMR of *Z*-**M1** (CD_2_Cl_2_, −40 °C): (red) stable-*Z*-**M1**, before irradiation (λ ≥ 365 nm); (green) after irradiation; and (blue) after standing at room temperature in the dark for 2 h.

The light-driven molecular motor **M1** employed in this study consists of two crucial parts: (i) an overcrowded-alkene-based central core as the rotary part and (ii) two acrylate moieties for copolymerization in the liquid crystal network (Figure 2A). In the present study, we chose a second-generation rotary motor with a cyclopentene upper- and a fluorenene-lower half (Figure 2A) as motors of similar structures have rotary speeds around 1 min at rt,^65,74^ which fits our purpose of constructing a fast responsive system. A C-6 carbon spacer was installed between the motor core and the acrylate groups to provide enough free space for the motor to rotate inside the polymer network. The designed molecular motor **M1** can undergo a full 360-degree rotary cycle of the upper rotor part with respect to the lower stator part, with the central olefinic double bond functioning as the rotational axle (Figure 2A). The four-step rotary cycle contains two phototriggered isomerization processes (Figure 2A, steps 1 and 3) around the central olefinic bond, each followed by a thermal helix inversion (THI) step (Figure 2A, steps 2 and 4).

### ^1^H NMR Studies

The synthesis of **M1** and separation of enantiomeric pure (*R*)-**M1** and (*S*)-**M1** are detailed in the SI. The assignment of *Z* and *E* isomers of **M1** is based on comparison with the related second-generation motor and specifically the chemical shift of the proton H_a_, which appears as a singlet at the lower half. As H_a_ of the *Z* isomer is in closer proximity with the xylene moiety of the upper half, it shifts more upfield when compared to that of the *E* isomer. We then assigned the isomer with H_a_ appearing at 7.40 ppm as the *E* isomer, while the isomer with H_a_ appearing at 6.90 ppm was considered to be the *Z* isomer (Figures S18 and S20). ^1^H NMR studies were subsequently employed to follow the isomerization process involved in the rotary of **M1**, starting from the stable-*E*-**M1**.

Figure 2B (red) displays a partial ^1^H NMR spectrum of stable *E* isomers in the CD_2_Cl_2_ solution. Distinctive features of the motor moiety are the signals of the aliphatic protons H_b_, H_c_, and H_d_ and the protons of the Me group at the stereogenic center. The doublet at 2.55 ppm is considered to be proton H_b_ as only a negligible coupling is usually observed between H_b_ and H_d_ due to their relative orientations as a result of the conformation of the five-membered ring. In addition, the double doublet at 3.16 ppm can be assigned to H_c_ as H_c_ couples not only to its geminal proton H_b_ but also to the vicinal proton H_d_. The multiplet at 3.94 ppm is assigned as proton H_d_ as a result of coupling with the protons of the methyl group and the proton H_c_. Furthermore, the doublet at 1.29 ppm is considered to be the methyl group at the stereogenic center. The sample was then irradiated (λ = 365 nm) at −40 °C, and distinct changes were observed in the spectrum, which indicates the formation of a new isomer that was identified as unstable-*Z*-**M1** (Figure 2B, green). Proton H_b_ was observed to shift from 2.55 ppm (doublet) to 2.81 ppm (double doublet) because unstable-*Z*-**M1** adopts a different conformation from that of stable-*E*-**M1**, which allows the coupling between H_b_ and H_d_. The new absorptions at 3.36 and 6.80 ppm were assigned to H_c_ and H_a_ of unstable-*Z*-**M1**, respectively. Furthermore, the signal of the methyl group was observed to shift from 1.29 to 1.52 ppm, which indicates the conformational change of the methyl group from a pseudo-axial orientation in the stable isomer to a pseudo-equatorial orientation in the unstable isomer. The photostationary state (PSS) was obtained when no significant changes were observed in the spectrum after extended irradiation. The ratio was determined to be 85:15 (unstable-*Z*-**M1**/stable-*E*-**M1**) by integration of the signals for proton H_b_ in the stable isomer and the unstable isomer. Keeping the sample in the dark at room temperature for 2 h resulted in further changes of the spectrum (Figure 2B, blue), indicating the occurrence of the thermal helix inversion to convert unstable-*Z*-**M1** to stable-*Z*-**M1**. The signal of H_a_ in the unstable-*Z*-**M1** shifted downfield from 6.80 ppm to 7.00 ppm, which indicates the formation of stable-*Z*-**M1**. Notably, the ratio of stable-*Z*-**M1**/stable-*E*-**M1** (85:15) is equivalent to the ratio of unstable-*Z*-**M1**/stable-*E*-**M1**. It confirms the unidirectionality of the thermal isomerization of unstable-*Z*-**M1**, indicating the absence of the thermal *E*-*Z* isomerization in accordance with our previous observation of motors with similar structures.^63^ Another set of photochemical and thermal helix inversion steps completes the 360° rotary cycle (Figure 2C). The sample of stable-*Z*-**M1** (Figure 2C, red) was irradiated (λ = 365 nm) at −40 °C, and similar changes in spectra and PSS ratios (unstable-*E*-**M1**/stable-*Z*-**M1**, 85:15) were found (Figure 2C, green). Keeping the sample under exclusion of light led to the formation of stable-*E*-**M1** (Figure 2C, blue). The signal of H_a_ in the stable-*Z*-**M1** shifted downfield from 6.90 to 7.24 ppm and further to 7.46 ppm during the process (Figure 2C), which suggests the rotary motion from stable-*Z*-**M1** to stable-*E*-**M1**.

### UV–Vis Spectroscopy and CD Studies

In addition, UV–vis and CD spectroscopies were employed to study the rotary motion of motor **M1** as well. The UV–vis absorption spectra of stable-*E*-**M1** and stable-*Z*-**M1** in CH_2_Cl_2_ at 253 K both show absorption bands centered at 380 nm (Figure S24B,C, black lines). Irradiation of both samples with UV light (λ_max_ = 365 nm) resulted in a red shift of the bands at 380 nm to an absorption centered at 410 nm, indicating the photochemically induced formation of the unstable isomers (Figure S24B,C, red lines). Leaving the samples in the dark gave rise to the occurrence of the thermal helix inversion step resulting in the formation of the corresponding stable isomer, and the original UV–vis spectra were regained. The kinetic studies (Figure S25A,C) at different temperatures provided the rate constants of the first-order thermal isomerization process, and the Gibbs energies of activation based on the Eyring analysis (Figure S25B,D) were 84.45 kJ·mol^–1^ for the THI from the unstable-*Z* to the stable-*Z* isomer and 84.51 kJ·mol^–1^ for the THI from the unstable-*E* to the stable-*E* isomer. The half-lives (*t*_1/2_) were calculated to be 127.1 and 130.1 s, respectively, for the unstable-*Z* and unstable-*E* isomers at room temperature. These values are similar to those obtained from structurally related motors and are considered to have a high rotary speed.^63,64^ Furthermore, circular dichroism (CD) was used to characterize the distinct inherent helical chiral stereoisomers and confirm the unidirectional rotary motion of motor **M1**. (*S*)-Stable-*E*-**M1** displays a negative CD absorption at 380 nm (Figure S24D, black line), and upon irradiation with UV light, a new positive CD band is observed at 410 nm (Figure S24D, red line), which indicates the change of molecular helicity due to the formation of the unstable-*Z*-**M1** isomer (Figure S24A, step 1). An independent study of enantiomer (*R*)-**M1** showed similar but inverse CD effects (Figure S24E). The samples were kept in the dark at rt, and the original spectra were regained as a result of the thermal helix inversion step (Figure S24A, step 2 or 4). The combined spectroscopic data confirm that motor **M1** undergoes unidirectional motion at a fast speed at rt upon irradiation, which sets a solid base for the next step, i.e., functioning as a photoactuator in liquid crystal networks.

### Fast Wavy Motion of LCN by Racemic Motors

**Figure 3:**
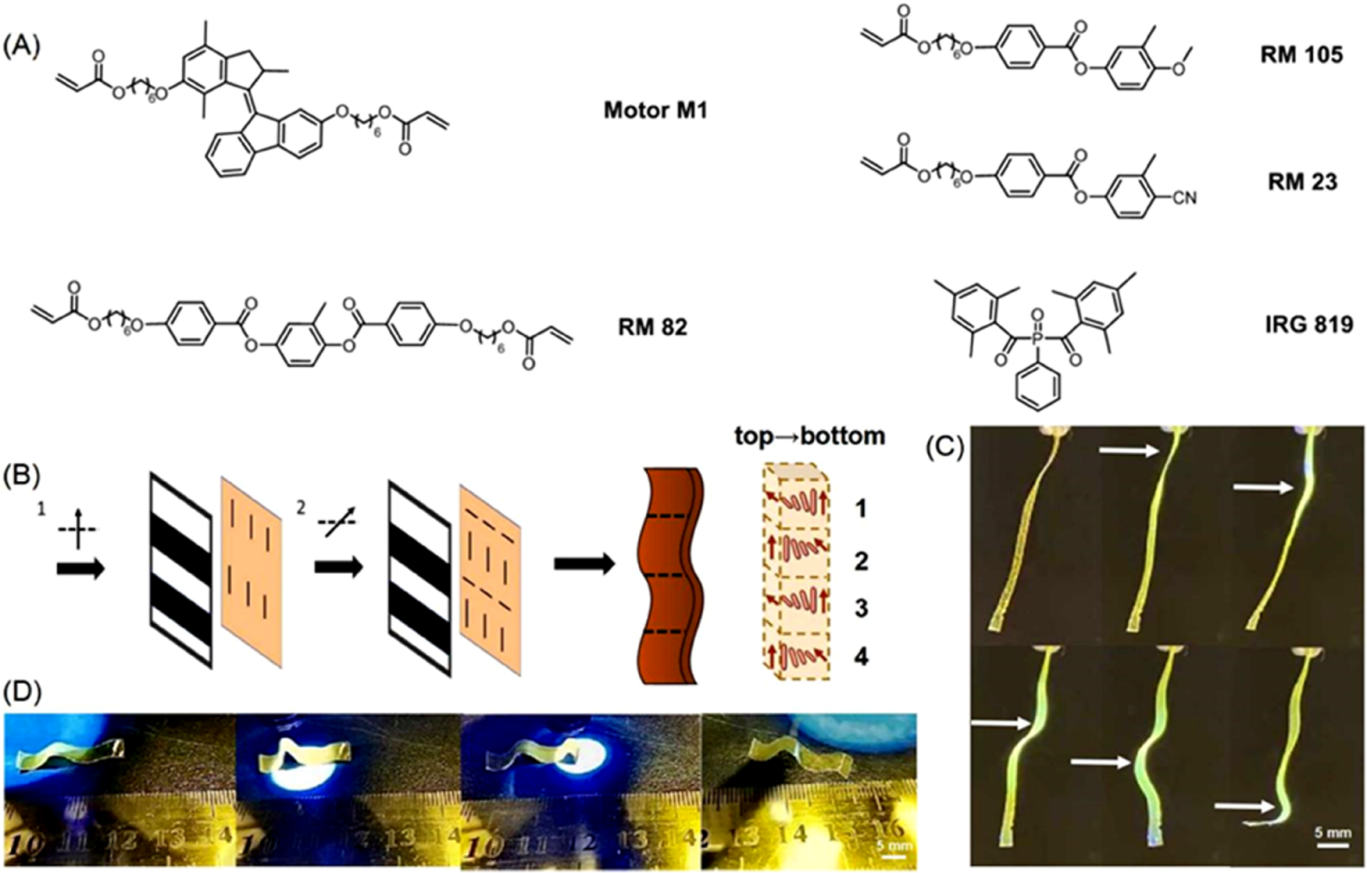
(A) Chemical structures of **M1**, liquid crystal mixtures (RM 82, RM 105, RM 23), and the photoinitiator (IRG 819). (B) Two-step procedure for the preparation of the alignment layers. The black arrows indicate the polarization direction of the UV light. Before the second exposure step, the sample is rotated at 90°. (C) Phototriggered wavy motion of the polymeric liquid crystal film. (D) Phototriggered translational movement of a piece of the polymeric LC film on a rough surface. The UV-light intensity is 100 mw/cm^2^.

With the confirmation of the rotary motion of **M1** in solution, the application of both racemic and homochiral motors as a cross-linker and an actuator for the liquid crystal network was subsequently studied. We first examined the racemic motor **M1** in combination with a mixture of LC monomers (RM 23, RM 82, RM 105, and IRG 819) (Figure 3A). The designed programmable polymeric ribbon is divided into four parts, and each part was a twisted alignment (Figure 3B). In the first part, the liquid crystal mixtures containing motor **M1** are aligned from a perpendicular to a parallel direction of the long axis of the ribbon from the top to the bottom. In the second part, the mixtures are aligned in a reversed manner, i.e., from the parallel to the perpendicular direction of the long axis of the ribbon from the top to the bottom. We then repeated the same patterns for the third and fourth parts. By the above design, the ribbon contains four alternating orientations along its long axis, as shown in Figure 3B.

We envisioned that by irradiation, different parts of the ribbon bend toward or against the light depending on the orientation and therefore contribute to a wavy motion. Two 2 × 2 cm^2^ glass substrates were first spin-coated with a photoalignment agent (SD1). Then, the substrates were exposed to UV linear polarized light under a photo mask (Figure 3B); layers with the aligning director oriented parallel to the polarization direction of the UV light were then generated in the exposed parts of both substrates. Next, both glass substrates were rotated clockwise at 90°; the exposed parts were then covered, and the second exposure was carried out to the previously unexposed parts. After the two-step exposure, glass substrates with alternating perpendicular alignments were formed (Figure 3B). Then, the two resulting glass substrates were glued together with a certain gap (20 μm) perpendicularly to form a glass cell. A liquid crystal mixture with 3 wt % racemic molecular motor **M1** (Figure 3A) was then filled into the above cell at 60 °C by capillary suction. After the mixture was fully aligned at 40 °C, the sample was cured with 455 nm light for 5 min, followed by an annealing process at 125 °C for 10 min. The resulting film was cooled to rt, and the DSC measurement confirmed the successful preparation of the polymeric film (Figures S26 and S27). Ribbons with 3 mm width were cut along the axis, and the free-standing sample was then submitted to irradiation studies. To our delight, when a specific part of the ribbon was exposed under UV light, it bent toward or against the light source and recovered to its initial position instantaneously after switching off the light (Supporting Movie S1) as expected. The first and third parts of the ribbon bent toward the light, while the second and fourth parts bent against the light (Figure 3C, Movie S1). We monitored the UV–vis spectra of the polymeric film during the irradiation, and the absorption of the film showed a decrease at 380 nm with a concomitant increase at 430 nm (Figure S28A), which is similar to the change observed with the motors in solution (Figure S25). It indicates the rotary motion of the motor in the LC ribbon during the irradiation. The isosbestic point at 400 nm also indicates a selective unimolecular process. After switching off the light, the absorption band at 380 nm recovered to its original intensity, and the original spectra were regained. The photoactuation and recovery have been repeated several times, and the system operates without significant fatigue during the cycles (Figure S28A). The UV–vis experiment confirms that the observed actuation of the LCN ribbon containing motor **M1** is predominantly due to the rotation and change in shape of the motor. The rotational motion of motor **M1**, and its effect on the polymer main chains, reduces the order parameter of the mesogenic units, which results in shrinkage along the molecular direction and expansion orthogonal to the ribbon. We performed additional control experiments to further exclude the possibility that the actuation is due to a photothermal effect. We monitored the temperature of the ribbon with an infrared camera, and no significant increase of temperature was observed during the actuation (Figure S30). In addition, we performed the actuation experiment under water. The LC ribbon was able to actuate under water after UV irradiation (Figure S31). Furthermore, we prepared a control compound that contains the same motor core structure but with only one acrylate group. The control compound was copolymerized using the same conditions, and the resulting ribbon did not show actuation after UV irradiation (Figure S32). The above control experiments explicitly confirm that the actuation of the LC ribbon is due to the rotary motion of the motors inside. We next took advantage of the phototriggered wavy motion of the ribbon. A ribbon with 20 mm length and 5 mm width was placed on a rough surface; UV light was then subsequently illuminated from the front to the back. By alternatingly waving toward or against the surface, the ribbon was able to move forward unidirectionally with a speed of 3.5 cm/min for a 1 × 1 cm^2^ film (Figure 3D, Movie S2), which compares favorably with most of the single-wavelength-triggered moving systems.^59−61^

### Complex Helical Motion of LCN by Chiral Motors

**Figure 4:**
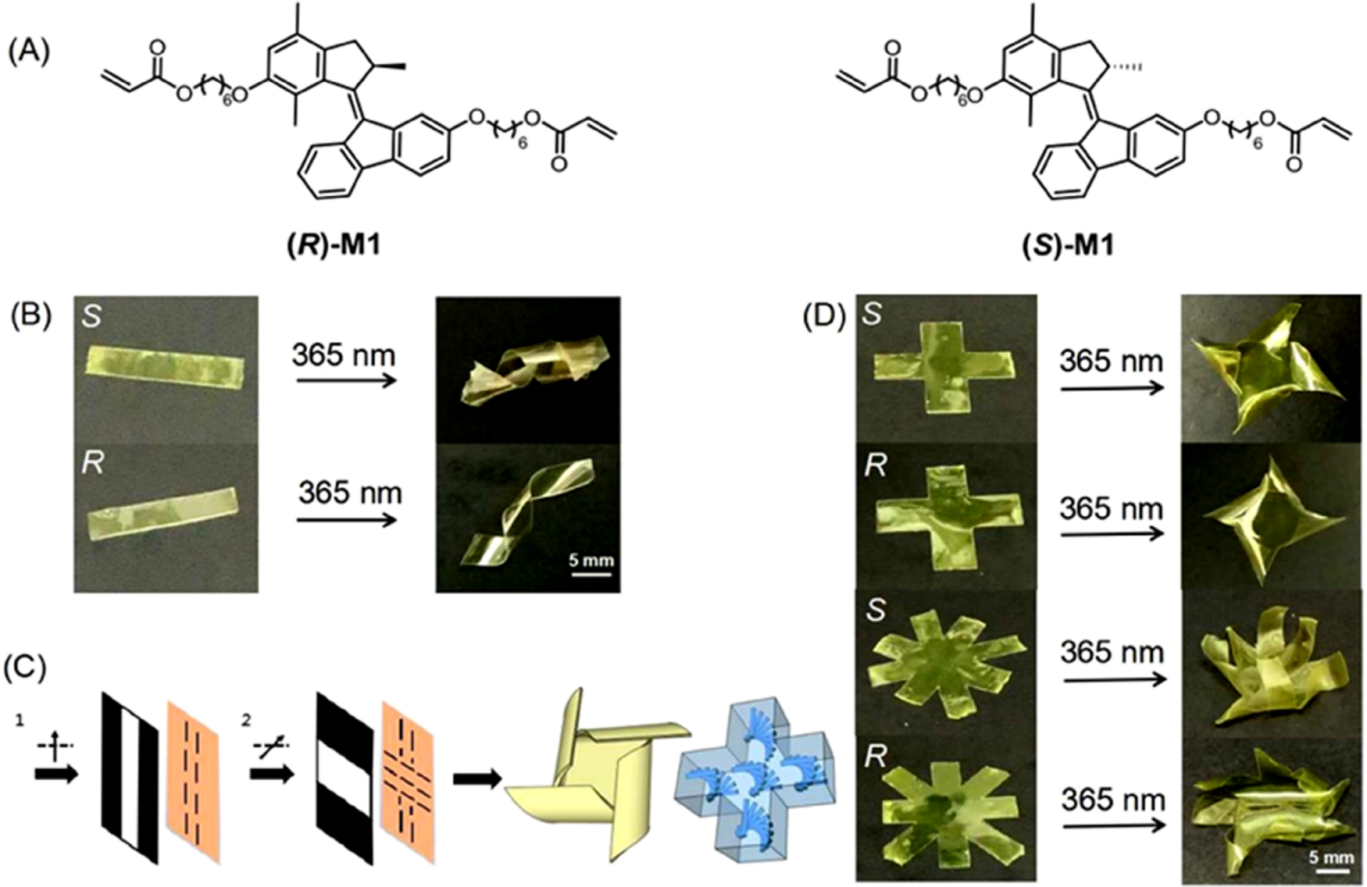
(A) Chemical structures of (*R*) and (*S*)-M1. (B) Phototriggered twisting of polymeric ribbons. When the ribbon contains the (*S*)-motor, the ribbon shows right-handed twisting, and vice versa. (C) Two-step procedure for the preparation of alignment layers. The black arrows indicate the polarization direction of the UV light. Before the second exposure step, the sample is rotated at 90°. (D) Phototriggered helical motion of the polymeric films with different shapes. The UV-light intensity is 230 mw/cm^2^.

Next, the enantiomerically pure motor **M1** was employed for the liquid crystal network (LCN). The molecular motor, owing to its special axial chirality, is an excellent chiral dopant to generate the cholesteric phase and therefore is able to induce helical motion in the LCN. In the present study, the helical twisting power of **M1** was determined to be ±115 μm^–1^, which suggests that **M1** is a strong chiral dopant.^75^ A mixture with 3 wt % enantiomeric motor **M1** (containing 1 wt % (*R*)- or (*S*)-**M1**) was filled into a cell with planar alignment layers (Figure 4A). As expected, the LC mixture formed a cholesteric phase in the cell (Figure S29). The samples were polymerized by exposure to UV light at 40°C for 5 min. The ribbon was cut along the orientation of the alignment layer. Gratifying, upon UV-light irradiation, ribbons that contain (*R*)-motors showed left-handed helical motion, while ribbons with (*S*)-motors displayed right-handed helical motion (Figure 4B, Movies S3 and S4). The ribbons reached their saturated states within 2 s and recovered to their original states after switching off the UV light. To achieve a more complex helical motion, a “programmable” alignment layer was prepared. Two glass substrates were exposed to UV linear polarized light under a photo mask. After the first exposure, the glass substrates were rotated clockwise with certain angles subsequently for the stepwise exposures (Figure 4C). It should be noted that the rotated angles define the shape change of the ribbon in the later stage. Then, the two resulting glass substrates were glued together with a fixed thickness of 50 μm to produce a cell with a planar alignment in certain directions. After the polymeric films were formed, they were cut along the defined directions of alignment layers. Ribbons with “cross” and “flower” shapes were obtained (for details of preparation of ribbons with different alignments and shapes, see the SI). To our delight, curly bending or folding motion of the ribbons was observed upon irradiation at 365 nm, with the wavelength inducing motor **M1** rotation and helicity change. As expected, the bending or folding direction is dependent on the handedness of the doped molecular motor (Figure 4D, Movies S5–S8).

**Figure 5:**
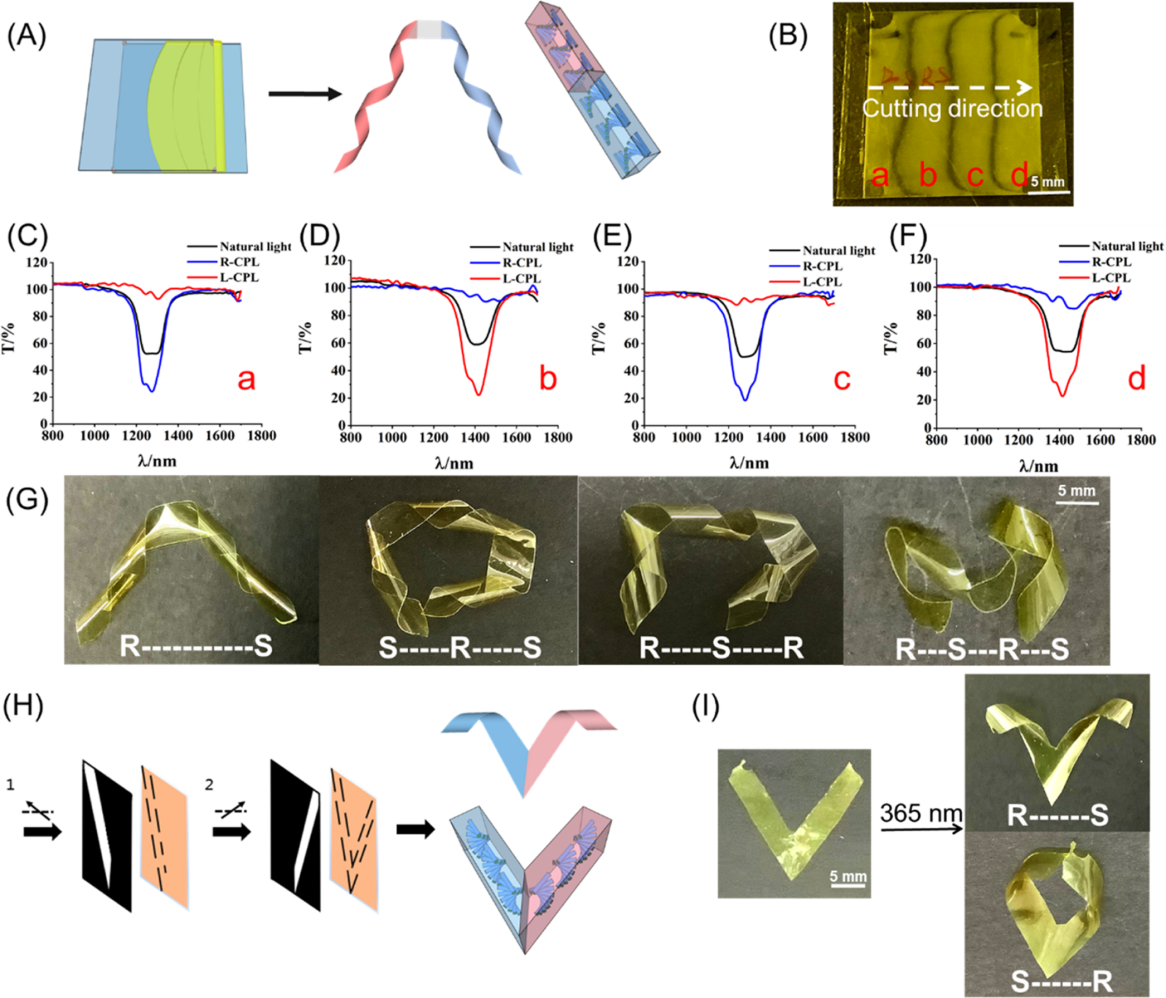
(A) Representative scheme for the preparation of the polymeric LC film with different handedness. (B) Preparation of the polymeric film with four stripes (a)–(d). Liquid crystal mixtures with the (*R*) or (*S*) motor (0.03 wt %) were filled alternatingly from the same side of a liquid crystal cell with the planar alignment at 80 °C. (C–F) Transmittance spectra of different stripes of the polymeric film. Stripes (a) and (c) showed 100% transmittance of the left-handed CPL, while stripes (b) and (d) showed 100% transmittance of the left-handed CPL. (G) Synchronized left–right- left-right–left-, right–left-right-, and left-right–left-right-handed motion in one single ribbon by single-wavelength irradiation. (H) Two-step procedure for the preparation of the alignment layers. The black arrows indicate the polarization direction of the UV light. (I) The resulting “V”-shaped film showed different helical motion upon light irradiation. The UV-light intensity is 230 mw/cm^2^.

We then examined the possibility of achieving multiple different helical motions in one single ribbon (Figure 5A) mimicking natural tendrils, which usually perform such sophisticated synchronized helical motion to support themselves. Liquid crystal mixtures with the (*R*) or (*S*) motor (0.03 wt %) were filled alternatingly from the same side of a liquid crystal cell with the planar alignment at 80 °C (Figure 5B). Circular polarized light (CPL) was then employed to trace whether diffusion of mixtures between each part happens. Figure 5C–F shows transmittance of different stripes in the LC sample (Figure 5B, a–d). When left-handed CPL was placed on stripe **a**, which contains the *R*-motor, it showed 100% transmittance, while the right-handed CPL showed only 20% transmittance (Figure 5C). It indicates that stripe **a** contains predominantly the (*R*)-motor. On the contrary, stripe **b** with the (*S*)-motor showed 100% transmittance of the right-handed CPL and 20% transmittance of the left-handed CPL (Figure 5D). The above CPL experiments confirmed that diffusion between each part containing chiral motors with different handedness is not significant. We anticipate that it might be due to the high viscosity of the liquid crystal mixture, and thus, diffusion is not profound within a certain time scale. After confirming the alternating helical structures of the cholesteric phase, the mixture was polymerized and the film was cut along the short axis of the stripes. Ribbons with the orders of (*R*)-(*S*), (*S*)-(*R*)-(*S*), (*R*)-(*S*)-(*R*), and (*R*)-(*S*)-(*R*)-(*S*) motors were obtained. Upon UV-light irradiation, multiple helical motions were observed at different areas of the ribbons. Figure 5G shows that, for the first time, synchronized left–right-, left-right–left-, right–left-right-, and left-right–left-right-handed motion can be achieved in one single ribbon by single-wavelength irradiation. In addition, we employed photolithography to construct a “V”-shaped polymeric ribbon containing motors with different handedness. Glass substrates were exposed to UV linear polarized light with a photo mask. After the first exposure, the glass substrates were rotated clockwise at 60° subsequently for the second exposure (Figure 5H). The two resulting glass substrates were glued together with a fixed thickness of 50 μm to produce a cell with planar alignment in ordered directions. Liquid crystal mixtures containing motors with different chiralities were filled into the cell alternatingly at 80 °C, and the cell was then cooled to 40 °C. Samples were exposed to UV light at 40 °C for 5 min. After the polymeric films were formed, they were cut along the “predefined” directions of alignment layers. The V-shaped polymer film undergoes “inward” or “outward” motions selectively depending on the (*R*)-(*S*) or (*S*)-(*R*) combination of the embedded molecular motor **M1** (Figure 5I, Movies S10 and S11).

## Conclusions

Realization of molecular systems that can undergo diverse and complex motions in a controlled and tunable way is a major challenge, and we show here that the key is to achieve effective organization of molecular machines in a well-defined environment and dynamic chirality is a distinct element that helps in cooperative amplification and directional motions along all length scales. In the present study, we report the programmable fabrication of liquid crystal polymer networks based on a light-driven rotary molecular motor. The rotary motion of the designed light-driven molecular motor was fully characterized by a variety of spectroscopic techniques prior to application in LCN. Both racemic and enantiomerically pure motors were copolymerized with LC monomers. We employed photolithography to preorder the alignment layers of the LC substrates, and therefore, molecular motors are within a controlled and well-defined orientation in the polymeric LC films. The motor unit has multiple functions, i.e., a cross-linker for the LC network, an intrinsic chiral dopant, and photoresponsive units to allow autonomous motion upon irradiation with a single wavelength of light. The obtained LC film containing racemic motors can perform light-triggered bending, wavy motion, and fast movement on surfaces. The film embedded with enantiomerically pure motors was able to achieve synchronized helical motion with different handedness. This study demonstrates how rotary motion of molecular motors can be programmed in light-responsive materials and eventually paves the way toward the design of advanced responsive and adaptive soft materials and inducing complex motion.

## Supporting Information Available

Transitional, bending, and complex helical motion of liquid crystal ribbons (MP4)
(MP4)
(MP4)
(MP4)
(MP4)
(MP4)
(MP4)
(MP4)
(MP4)
(MP4)
(MP4)
Experimental procedures and characterization data for all new compounds, UV–vis and CD studies, and DSC study (PDF)
