# Supplementary material for: Phototriggered Complex Motion by Programmable Construction of Light-Driven Molecular Motors in Liquid Crystal Networks
Source: J Am Chem Soc. 2022 Apr 5;144(15):6851–60. doi: 10.1021/jacs.2c01060 (PMC9026258; doi:10.1021/jacs.2c01060)
Supplement: Supplementary file 12 — ja2c01060_si_012.pdf [file ja2c01060_si_012.pdf]

**Supplementary Information for**

# Photo-triggered complex motion by programmable construction of light-driven molecular motors in liquid crystal networks

Jiaxin Hou[1,2, ‡], Guiying Long[1, ‡], Wei Zhao[3], Guofu Zhou[1,3], Danqing Liu[3,4], Dirk J. Broer[3,4], Ben L. Feringa*[,1,2], Jiawen Chen*[,1]

1 SCNU-UG International Joint Laboratory of Molecular Science and Displays, National Center for International Research on Green Optoelectronics, South China Normal University, Guangzhou 510006, China.

2 Stratingh Institute for Chemistry, University of Groningen, Nijenborgh 4, 9747AG Groningen, The Netherlands.

3 SCNU-TUE Joint lab of Device Integrated Responsive Materials (DIRM), Guangdong Provincial Key Laboratory of Optical Information Materials and Technology & Institute of Electronic Paper Displays, South China Academy of Advanced Optoelectronics, South China Normal University, Guangzhou 510006, China.

4 Stimuli-responsive Functional Materials and Devices, Department of Chemical Engineering and Chemistry, Eindhoven University of Technology, Den Dolech 2, Eindhoven, 5600 MB, The Netherlands.

‡J.-X.H and G.-Y.L contributed equally

Correspondence to Jiawen Chen and Ben L. Feringa

**General remark:** All chemicals were purchased from Sigma-Aldrich. All organic solvents were analytically pure, and dried or redistilled before use. For column chromatography, silica gel (Silicycles Siliaflash P60, 40–60 μm, 230–400 mesh) was used. Separation was carried out on silica gel 60 (silicon dioxide, $SiO_2$; Merck, Germany) and kieselguhr F254 (celite; Merck, Germany) for thin-layer chromatography (TLC), and visualization was accomplished by stain.

**Characterization:** $^1$H-NMR and $^{13}$C-NMR spectra were recorded on a Varian AMX-500 (500 MHz)or a Varian AMX-400 (400 MHz). Irradiation studies were recorded on a Varian AMX-500 (500 MHz) in dichloromethane-$d_2$ ($CD_2Cl_2$). The corresponding chemical shifts were reported in δ values (ppm) relative to deuterochloroform ($CDCl_3$; $^1$H δ=7.25, $^{13}$C δ=77.2, $CD_2Cl_2$; $^1$H δ=5.32, $^{13}$C δ=54). For $^1$H-NMR, the signals were assigned as following: singlet (s), doublet (d), double doublet (dd), triplet (t), quartet (q) and multiplet (m). Proton magnetic resonance spectroscopy (HRMS) was measured using a double focusing high-resolution mass spectrometer (MS-902, AEI). Ultraviolet-visible (UV-vis) spectra were obtained with HP8454 UV-Vis spectrophotometer in a 1 cm quartz cuvette at room temperature. Solution circular dichroism (CD) spectra were recorded on a JASCO J-715 spectropolarimeter at room temperature. Irradiation experiments were performed using an LED lamp (Thorlabs) at 365 nm. All the optical phenomena of CLC mixture were observed and recorded via polarizing optical microscope (POM; DM2700p, Leica). Enantiopure motor was obtained by chiral HPLC (ODH; isopropanol: heptane= 1:99, flow rate 1 mL/min).

## Synthetic Route:

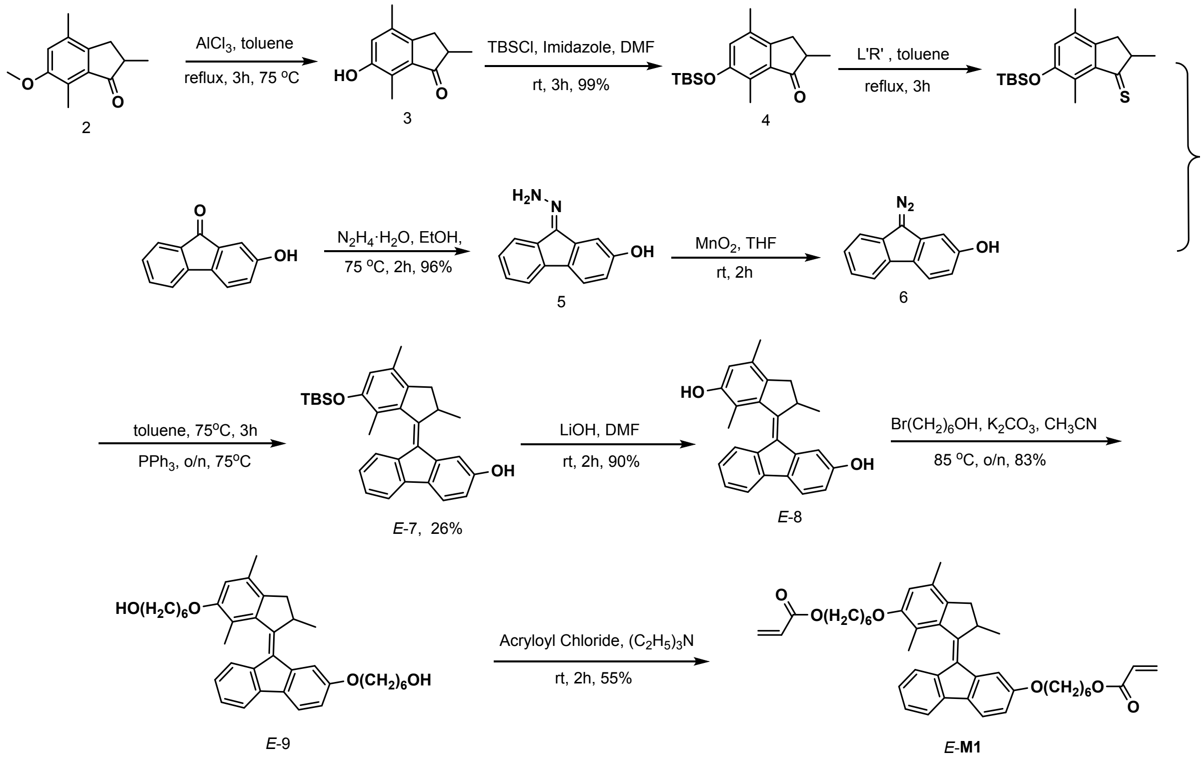


**Figure S1.** Light-driven rotary molecular motors. Synthetic route of ***E*-M1**.

### 6-methoxy-2,4,7-trimethyl-2,3-dihydro-1H-inden-1-one (2)

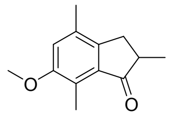

**Compound 2** was synthesized following the procedure in ref 1.$^{1}$H NMR (300 MHz, Chloroform-d) δ 6.91 (s, 1H), 3.83 (d, J = 0.9 Hz, 3H), 3.15 (dd, J = 16.7, 8.0 Hz, 1H), 2.65 (m, J = 7.3, 3.9 Hz, 1H), 2.49 (s, 3H), 2.43 (d, J = 4.1 Hz, 1H), 1.28 (dd, J = 7.4, 0.9 Hz, 3H).

### 6-hydroxy-2,4,7-trimethyl-2,3-dihydro-1H-inden-1-one (3)

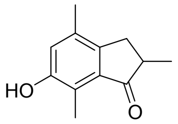

**Compound 3** was synthesized following the procedure in ref 1. $^{1}$H NMR (400 MHz, Chloroform-d) δ 6.89 (s, 1H), 3.13 (dd, J = 16.7, 7.9 Hz, 1H), 2.65 (m, J = 7.5, 3.9 Hz, 1H), 2.50 (s, 3H), 2.43 (dd, J = 16.7, 3.9 Hz, 1H), 2.23 (s, 3H), 1.27 (d, J = 7.4 Hz, 3H).

### 6-((tert-butyldimethylsilyl)oxy)-2,4,7-trimethyl-2,3-dihydro-1H-inden-1-one (4)

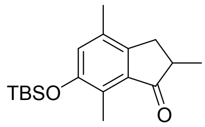

To a mixture of **3** (0.37 g, 2.10 mmol) and imidazole (0.50 g, 7.10 mmol) in dry DMF (20 mL), *tert*-butyldimethylsilyl chloride (0.86 g, 5.68 mmol) was added. The mixture was stirred at room temperature overnight under nitrogen, then quenched with water. The mixture was extracted with EtOAc (2 × 100 mL). The organic layer was washed with brine and dried over $Na_2SO_4$. The solvent was removed in vacuo. The residue was purified by flash column ($SiO_2$, pentane: EtOAc=8:1) to yield **5** (0.63 g, 2.1 mmol, 99%) as a colorless oil. $^{1}$H NMR (600 MHz, Chloroform-d) δ 6.85 (s, 1H), 3.17 (dd, J = 16.7, 7.9 Hz, 1H), 2.68 (m, J = 7.5, 4.0 Hz, 1H), 2.49 (s, 3H), 2.46 (d, J =

4.0 Hz, 1H), 2.26 (s, 3H), 1.31 (d, J = 7.4 Hz, 3H), 1.05 (s, 9H). $^{13}C$ NMR (151 MHz, Chloroform-d) δ 211.0, 153.0, 145.9, 134.6, 132.6, 125.9, 77.2, 77.0, 76.8, 42.7, 32.6, 25.8, 18.3, 17.6, 16.6, 10.7. HRMS (ESI) m/z calculated for $[M+H]^+$ $C_{18}H_{28}O_2Si$: 305.1931, found: 305.1945.

**(2-((tert-butyldimethylsilyl)oxy)-9H-fluoren-9-ylidene)hydrazine (5)**

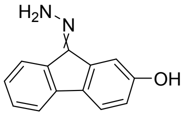


**Compound 5** was synthesized following the procedure in ref 2. $^1H$ NMR (600 MHz, DMSO-$d_6$) δ 9.62 (s, 1H), 9.50 (s, 1H), 8.09 (d, *J* = 7.6 Hz, 1H), 7.94 (s, 1H), 7.84 (s, 2H), 7.72 (d, *J* = 7.5 Hz, 1H), 7.69 (d, *J* = 8.1 Hz, 1H), 7.64 (dd, *J* = 7.5, 0.9 Hz, 1H), 7.60 – 7.58 (m, 1H), 7.58 – 7.54 (m, 1H), 7.37 – 7.33 (m, 1H), 7.25 (dtd, *J* = 7.4, 3.7, 1.2 Hz, 2H), 7.17 (td, *J* = 7.4, 1.1 Hz, 1H), 7.03 (d, *J* = 2.2 Hz, 1H). $^{13}C$ NMR (151 MHz, DMSO-$d_6$) δ 158.0, 158.0, 131.6, 128.9, 127.6, 126.3, 126.3, 125.4, 121.4, 121.2, 119.8, 119.5, 119.0, 115.6, 115.1, 113.4, 106.7. HRMS (ESI) calculated for $C_{13}H_{10}N_2O$ 211.0827 (M+), found 211.0829.

**(*E,Z*)-9-(6-((tert-butyldimethylsilyl)oxy)-2,4,7-trimethyl-2,3-dihydro-1H-inden-1-ylidene)-9H-fluoren-2-ol (7)**

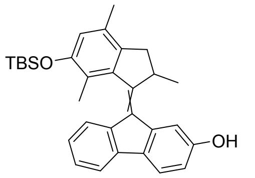


To a solution of **4** (0.50 g, 1.52 mmol) in dry toluene (50 mL), Lawesson's reagent (1.39 g, 3.44 mmol) was added. The mixture was stirred at reflux for 2 h. After the reaction finished, toluene was removed under reduced pressure. The residue was purified by flash column ($SiO_2$, pentane: EtOAc=10:1) to obtain a purple solution of thioketone**.** The solvent was removed under reduced pressure to obtain thioketone as purple solid. Thioketone was immediately added to a solution of diazo compound **6** in dry THF (10 mL). The diazo compound was prepared by mixing $MnO_2$ (1.20 g, 13.40 mmol) and hydrazone **5** (0.70 g, 2.16 mmol) in THF (10 mL) for 2 h followed by a filtration to remove the $MnO_2$. The diazo and thioketone mixture was stirred at 75 °C for 3 h. After 3 h, $PPh_3$ (0.70 g, 2.60 mmol) was added to the mixture. The mixture was stirred at 75 °C overnight and the solvent was removed in vacuo. The residue was purified by flash column ($SiO_2$, pentane: EtOAc=10:1) to yield motor *E*-**7** (0.25 g, 0.44 mmol, 26%) and motor *Z*-**7** (0.17 g, 0.30 mmol, 18%) as a yellow solid.

***E*-7**: $^1H$ NMR (400 MHz, Chloroform-*d*) δ 7.62 (dd, *J* = 7.90, 4.75 Hz, 2H), 7.39 – 7.31 (m, 2H), 7.27 – 7.21 (m, 2H), 7.01 (td, *J* = 7.59, 1.23 Hz, 1H), 6.83 (m, *J* = 8.16, 3.82, 2.29 Hz, 1H), 6.68 (s, 1H), 4.06 (t, *J* = 6.47 Hz, 1H), 3.11 (dd, *J* = 14.53, 5.78 Hz, 1H), 2.49 (d, *J* = 14.57 Hz, 1H), 2.25 (d, *J* = 20.93 Hz, 3H), 2.08 (s, 3H), 1.04 (s, 10H), 0.28 (s, 6H), 0.22 (d, *J* = 3.87 Hz, 2H). $^{13}C$ NMR (101 MHz, Chloroform-d) δ 157.7, 155.7, 143.9, 142.0, 140.7, 140.3, 136.0, 134.7, 129.7, 128.7, 128.0, 128.0, 127.0, 126.5, 124.0, 123.0, 120.9, 116.7, 113.9, 46.7, 42.0, 28.6, 28.5, 28.5, 21.7, 21.1, 21.0, 19.1, 15.8. HRMS (ESI) m/z calculated for $[M+H]^+$ $C_{31}H_{36}O_2Si$: 469.2557, found: 469.2557.

***Z*-7** : $^1H$ NMR (400 MHz, Chloroform-d) δ 7.86 – 7.79 (m, 1H), 7.70 – 7.64 (m, 1H), 7.56 (d, J = 8.14 Hz, 1H), 7.28 (m, J = 18.64, 7.36, 1.27 Hz, 2H), 6.83 (d, J = 2.32 Hz, 1H), 6.76 (dd, J = 8.14, 2.33 Hz, 1H), 6.67 (s, 1H), 4.10 (m, J = 6.67, 3.76, 2.75 Hz, 1H), 3.12 (dd, J = 14.53, 5.81 Hz, 1H), 2.50 (d, J = 14.46 Hz, 1H), 2.25 (s, 3H), 2.10 (s, 3H), 1.28 (d, J = 6.72 Hz, 3H), 1.04 (s, 9H), 0.28 (d, J = 8.04 Hz, 5H). $^{13}C$ NMR (101 MHz, Chloroform-d) δ 157.9, 155.6, 155.4, 143.9, 142.7, 142.3, 142.1, 140.9, 135.5, 134.9, 129.5, 128.5, 128.4, 128.3, 126.5, 123.9,

122.6, 121.4, 117.1, 113.4, 63.2, 47.0, 42.0, 28.6, 28.5, 28.5, 23.7, 21.7, 21.2, 19.1, 16.8. HRMS (ESI) m/z calculated for $[M+H]^+$ $C_{31}H_{36}O_2Si$: 469.2557, found: 469.2576.

**(*E, Z*)-9-(6-hydroxy-2,4,7-trimethyl-2,3-dihydro-1H-inden-1-ylidene)-9H-fluoren-2-ol (8)**

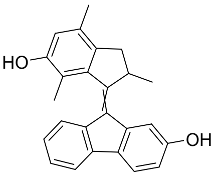


To a solution of motor ***E*-7** (0.33g, 0.70mmol) in DMF (10 mL), LiOH (0.084 g, 3.50 mmol) was added. The mixture was stirred at room temperature for 2 h and quenched with water. The pH value of the mixture was adjusted to 6 with HCl (1 M, aq) solution. The water phase was extracted with EtOAc (2 × 150 mL). The combined organic layers were washed with brine and dried over $Na_2SO_4$. The residue was purified by flash column ($SiO_2$, pentane: EtOAc=1:1) to yield ***E*-8** (0.22 g, 0.63 mmol, 90%) as yellow solid.

***Z*-8** was synthesized with the same procedure as ***E*-8.**

***E*-8 :** $^1$H NMR (400 MHz, DMSO-*d*$_6$) δ 9.52 (s, 1H), 9.20 (s, 1H), 7.64 (dd, *J* = 7.85, 4.80 Hz, 2H), 7.24 – 7.10 (m, 2H), 7.05 – 6.88 (m, 1H), 6.76 (dd, *J* = 8.20, 2.00 Hz, 1H), 6.69 (s, 1H), 3.93 (m, *J* = 6.08 Hz, 1H), 3.03 (dd, *J* = 14.59, 5.72 Hz, 1H), 2.17 (s, 3H), 1.92 (s, 3H), 1.19 (d, *J* = 6.58 Hz, 3H). $^{13}$C NMR (151 MHz, DMSO-*d*$_6$) δ 157.7, 155.0, 153.1, 141.1, 140.7, 139.9, 137.1, 135.7, 132.2, 131.3, 129.7, 127.6, 125.4, 123.6, 121.1, 120.8, 118.7, 117.4, 114.8, 111.4, 60.3, 19.5, 18.6, 16.0, 14.5. HRMS (ESI) m/z calculated for $[M+H]^+$ $C_{25}H_{22}O_2$: 355.1693, found: 355.1686.

***Z*-8 :** $^1$H NMR (400 MHz, DMSO-d6) δ 9.30 (s, 1H), 9.20 (s, 1H), 7.77 – 7.65 (m, 2H), 7.58 (d, J = 7.97 Hz, 1H), 7.24 (m, J = 18.61, 7.35, 1.25 Hz, 2H), 6.73 – 6.62 (m, 3H), 3.97 (m, J = 12.57, 6.47, 5.98 Hz, 1H), 3.02 (dd, J = 14.46, 5.67 Hz, 1H), 2.17 (s, 3H), 1.95 (d, J = 5.05 Hz, 3H), 1.18 (d, J = 6.63 Hz, 3H). $^{13}$C NMR (151 MHz, DMSO-*d*$_6$) δ 157.4, 155.1, 153.1, 140.6, 140.1, 139.4, 139.1, 135.7, 132.1, 131.2, 129.8, 127.4, 126.1, 124.0, 120.9, 120.5, 119.1, 117.3, 115.1, 111.1, 44.4, 39.3, 19.6, 18.6, 16.0. HRMS (ESI) m/z calculated for $[M+H]^+$ $C_{25}H_{22}O_2$: 355.1693, found: 355.1695.

**(*E,Z*)-6-((9-(6-((6-hydroxyhexyl)oxy)-2,4,7-trimethyl-2,3-dihydro-1H-inden-1-ylidene)-9H-fluoren-2-yl)oxy)hexan-1-ol (9)**

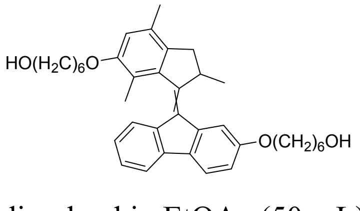


To a solution of ***E*-8** (0.1 g, 0.29 mmol) in $CH_3CN$ (10 mL), $K_2CO_3$ (0.20 g, 1.45 mmol), TBAI (0.086 g, 0.23 mmol) and 6-bromohexan-1-ol (0.15 mL, 1.16 mmol) were added. The mixture was stirred at 85 $^o$C under nitrogen overnight. The solvent was removed under reduced pressure, the residue was dissolved in EtOAc (50 mL) and organic layer was washed with water (2×100 mL) and brine, then dried over $Na_2SO_4$. The solvent was removed in vacuo and the residue was purified by flash column ($SiO_2$, pentane: EtOAc=1:3) to yield motor ***E*-9** (0.12 g, 0.22 mmol, 77%) as yellow solids.

***Z*-9** was synthesized with the same procedure as ***E*-9.**

***E*-9 :** $^1$H NMR (400 MHz, Chloroform-*d*) δ 7.63 (dd, *J* = 15.96, 7.91 Hz, 2H), 7.42 (d, *J* = 2.24 Hz, 1H), 7.32 (d, *J* = 7.87 Hz, 1H), 7.26 – 7.17 (m, 1H), 7.04 (m, *J* = 7.56, 1.13 Hz, 1H), 6.89 (dd, *J* = 8.32, 2.14 Hz, 1H), 6.70 (s, 1H), 5.27 (s, 3H), 4.11 (dd, *J* = 8.11, 4.64 Hz, 1H), 4.05 (m, *J* = 6.54, 5.23 Hz, 3H), 3.94 (m, *J* = 9.14, 6.56 Hz, 1H), 3.64 (td, *J* = 6.56, 2.88 Hz, 4H),

3.14 (dd, *J* = 14.52, 5.77 Hz, 1H), 2.88 (d, *J* = 22.41 Hz, 1H), 2.51 (d, *J* = 14.45 Hz, 1H), 2.29 (s, 3H), 2.10 (s, 3H), 1.80 (s, 0H), 1.78 (s, 1H), 1.66 – 1.28 (m, 9H). $^{13}$C NMR (151 MHz, Chloroform-*d*) δ 158.8, 156.7, 152.4, 141.2, 141.0, 139.5, 137.7, 136.6, 133.2, 131.8, 130.5, 127.0, 125.5, 123.9, 123.9, 120.1, 118.3, 113.6, 113.3, 110.9, 68.6, 68.3, 63.0, 44.2, 39.3, 32.7, 32.7, 29.5, 29.4, 26.1, 26.0, 25.6, 25.6, 19.1, 18.8, 15.7. HRMS (ESI) m/z calculated for $[M+H]^+$ $C_{37}H_{46}O_4$: 555.3469, found: 555.3487.

***Z*-9 :** $^{1}$H NMR (400 MHz, Chloroform-*d*) δ 7.81 (d, *J* = 7.45 Hz, 1H), 7.66 (dd, *J* = 7.24, 1.59 Hz, 1H), 7.57 (d, *J* = 8.25 Hz, 1H), 7.41 – 7.18 (m, 2H), 6.99 (d, *J* = 2.31 Hz, 1H), 6.82 (d, *J* = 8.32 Hz, 1H), 6.69 (s, 1H), 4.12 (dd, *J* = 12.43, 5.33 Hz, 2H), 3.99 (ddd, *J* = 9.67, 6.71 Hz, 2H), 3.87 – 3.71 (m, 2H), 3.62 (m, *J* = 10.13, 6.63 Hz, 4H), 3.13 (dd, *J* = 14.53, 5.87 Hz, 1H), 2.51 (d, *J* = 14.39 Hz, 1H), 2.28 (s, 3H), 2.21 – 2.11 (m, 4H), 1.83 (q, *J* = 7.00 Hz, 3H), 1.74 – 1.35 (m, 17H), 1.31 (d, *J* = 6.70 Hz, 3H). $^{13}$C NMR (151 MHz, Chloroform-*d*) δ 158.6, 156.7, 152.5, 140.9, 140.1, 139.5, 139.3, 136.6, 132.7, 131.9, 130.5, 126.9, 125.7, 123.9, 123.6, 119.7, 118.8, 114.8, 113.6, 109.3, 68.6, 67.7, 62.8, 44.1, 39.3, 32.7, 31.5, 30.2, 29.7, 29.4, 26.1, 25.9, 25.7, 25.6, 19.1, 18.8, 15.8. HRMS (ESI) m/z calculated for $[M+H]^+$ $C_{37}H_{46}O_4$: 555.3469, found: 555.3494.

**6-((9-(6-((6-(acryloyloxy)hexyl)oxy)-2,4,7-trimethyl-2,3-dihydro-1H-inden-1-ylidene)-9H-fluoren-2-yl)oxy)hexyl acrylate (M1)**

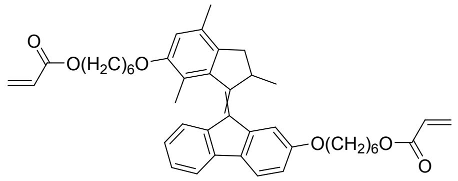


To a solution of ***E*-9** (0.10 g, 0.22 mmol) in THF (5 mL), triethylamine (0.61 mL, 4.4 mmol) and acryloyl chloride (0.04 mL, 0.49 mmol) were added at 0 °C. The mixture was allowed to warm up to room temperature and stirred for 2 h. The mixture was quenched with water (100 mL) and the aqueous phase was extracted with EtOAc (2 × 150 mL). The combined organic layer was washed with saturated $NH_4Cl$ solution (2 × 150 mL) and brine, then dried over $Na_2SO_4$. The solvent was removed in vacuo and the residue was purified by flash column ($SiO_2$, pentane: EtOAc=6:1) to yield ***E*-M1** as a yellow solid (0.07 g, 0.11 mmol, 50%). Enantiopure ***E*-M1** were obtained by chiral HPLC (ODH; isopropanol: heptane= 1:99, flow rate 1 mL/min).

***Z*-M1** was synthesized with the same procedure as ***E*-M1.**

***E*-M1 :** $^{1}$H NMR (300 MHz, Chloroform-*d*) δ 7.66 (dd, *J* = 12.36, 7.90 Hz, 2H), 7.45 (d, *J* = 2.21 Hz, 1H), 7.34 (d, *J* = 7.84 Hz, 1H), 7.25 (t, *J* = 7.40 Hz, 1H), 7.11 – 7.01 (m, 1H), 6.92 (dd, *J* = 8.37, 2.10 Hz, 1H), 6.73 (s, 1H), 6.41 (ddd, *J* = 17.30, 3.16, 1.53 Hz, 2H), 6.13 (ddd, *J* = 17.30, 10.36, 3.27 Hz, 2H), 5.82 (ddd, *J* = 10.36, 3.90, 1.58 Hz, 2H), 4.28 – 4.14 (m, 6H), 4.09 (q, *J* = 6.46 Hz, 4H), 3.96 (dd, *J* = 9.13, 6.52 Hz, 1H), 3.17 (dd, *J* = 14.47, 5.74 Hz, 1H), 2.54 (d, *J* = 14.42 Hz, 1H), 2.32 (s, 3H), 2.12 (s, 3H), 1.85 (m, *J* = 15.24, 8.67, 7.52 Hz, 5H), 1.74 (td, J = 6.73, 3.55 Hz, 5H), 1.63 – 1.41 (m, 11H), 1.35 (d, J = 6.64 Hz, 3H).$^{13}$C NMR (151 MHz, Chloroform-*d*) δ 166.4, 158.8, 156.7, 152.4, 141.3, 141.0, 139.5, 137.8, 136.7, 133.3, 131.8, 130.6, 130.6, 130.5, 128.6, 127.1, 125.5, 124.0, 123.9, 120.2, 118.3, 113.6, 113.3, 110.9, 68.6, 68.2, 64.6, 64.6, 44.2, 39.3, 29.5, 29.3, 28.7, 28.6, 26.0, 25.9, 25.8, 19.1, 18.8, 15.7. HRMS (ESI) m/z calculated for $[M+Na]^+$ $C_{43}H_{50}O_6$: 685.3500, found: 685.3529.

***Z*-M1 :** $^{1}$H NMR (400 MHz, Chloroform-*d*) δ 7.82 (d, *J* = 7.59 Hz, 1H), 7.67 (d, *J* = 7.08 Hz, 1H), 7.58 (d, *J* = 8.79 Hz, 1H), 7.27 (m, *J* = 21.55, 7.50 Hz, 2H), 7.00 (d, *J* = 2.20 Hz, 1H), 6.85 – 6.79 (m, 1H), 6.70 (s, 1H), 6.39 (d, *J* = 17.26 Hz, 2H), 6.11 (dd, *J* = 17.34, 10.39 Hz,

2H), 5.79 (d, *J* = 10.43 Hz, 2H), 4.14 (m, *J* = 15.76, 7.63, 6.93 Hz, 5H), 4.00 (dd, *J* = 21.79, 7.99, 7.04 Hz, 2H), 3.78 (m, *J* = 23.29, 8.36, 7.52 Hz, 2H), 3.14 (dd, *J* = 14.67, 5.68 Hz, 1H), 2.52 (d, *J* = 14.92 Hz, 1H), 2.30 (s, 3H), 2.15 (s, 2H), 1.83 (m, *J* = 7.08 Hz, 3H), 1.78 – 1.62 (m, 6H), 1.59 – 1.36 (m, 9H), 1.31 (d, *J* = 6.50 Hz, 3H). $^{13}$C NMR (151 MHz, Chloroform-*d*) δ 166.3, 166.3, 158.6, 156.7, 152.4, 140.9, 140.1, 139.5, 139.4, 136.6, 132.7, 131.9, 130.6, 130.6, 130.5, 128.6, 128.6, 126.9, 125.7, 123.9, 123.6, 119.7, 118.8, 114.8, 113.5, 109.3, 68.5, 67.7, 64.6, 64.6, 44.1, 39.3, 29.6, 29.2, 28.7, 28.6, 26.0, 25.9, 25.8, 25.7, 19.1, 18.8, 15.8. HRMS (ESI) m/z calculated for [M+Na]$^+$ $C_{43}H_{50}O_6$: 685.3500, found: 685.3495.

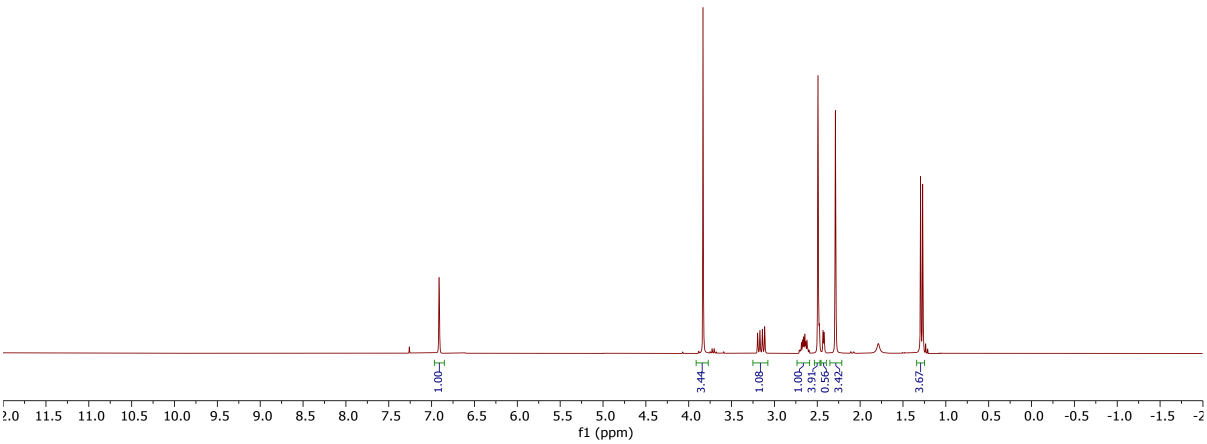


**Figure S2** $^1$H-NMR spectrum of **compound 2** in $CDCl_3$.

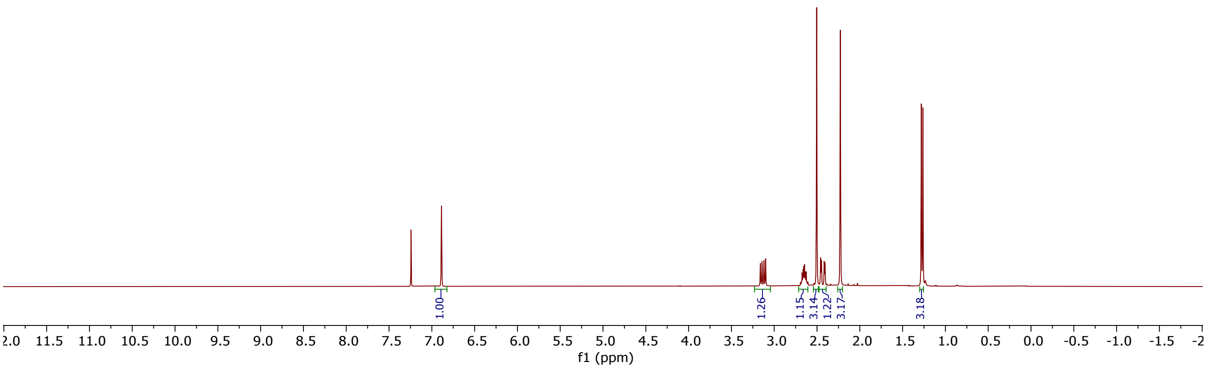


**Figure S3** $^1$H-NMR spectrum of **compound 3** in $CDCl_3$.

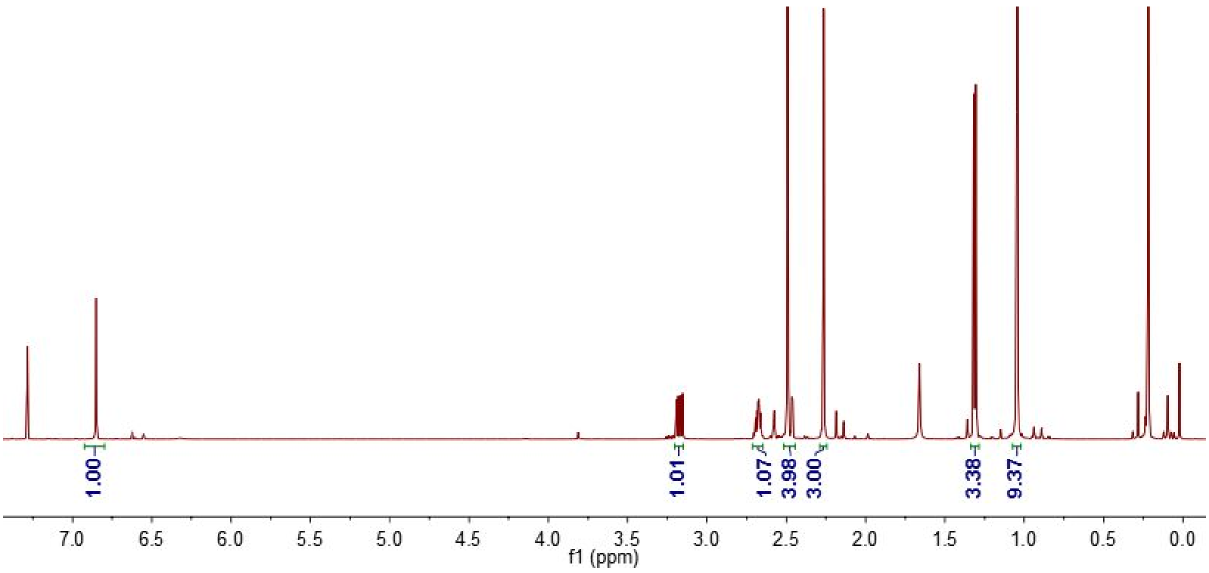


**Figure S4** $^{1}$H-NMR spectrum of **compound 4** in $CDCl_3$.

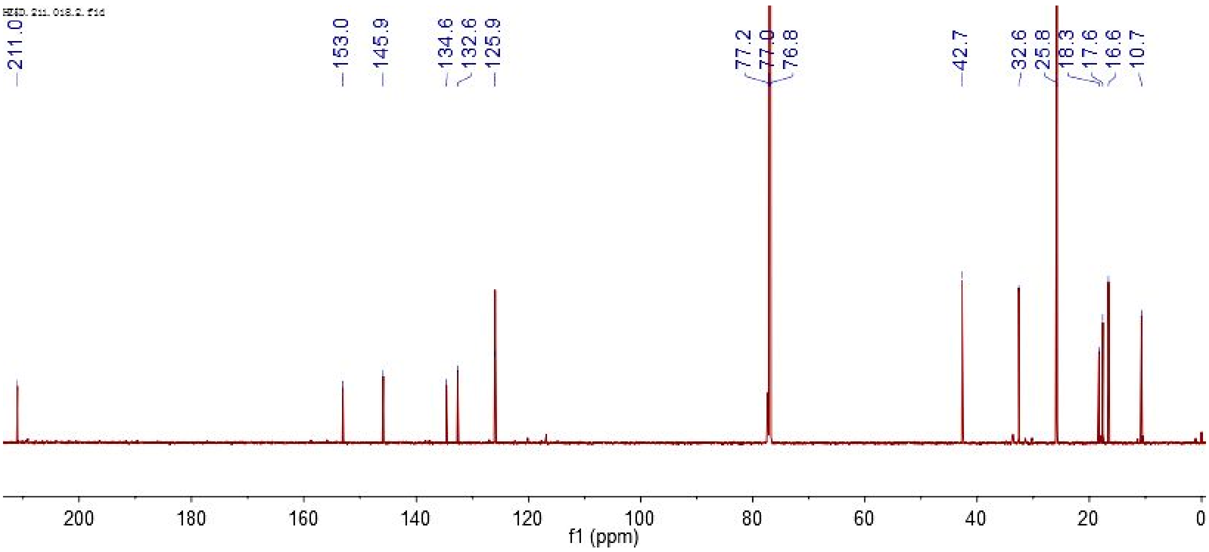


**Figure S5** $^{13}$C-NMR spectrum of **compound 4** in $CDCl_3$.

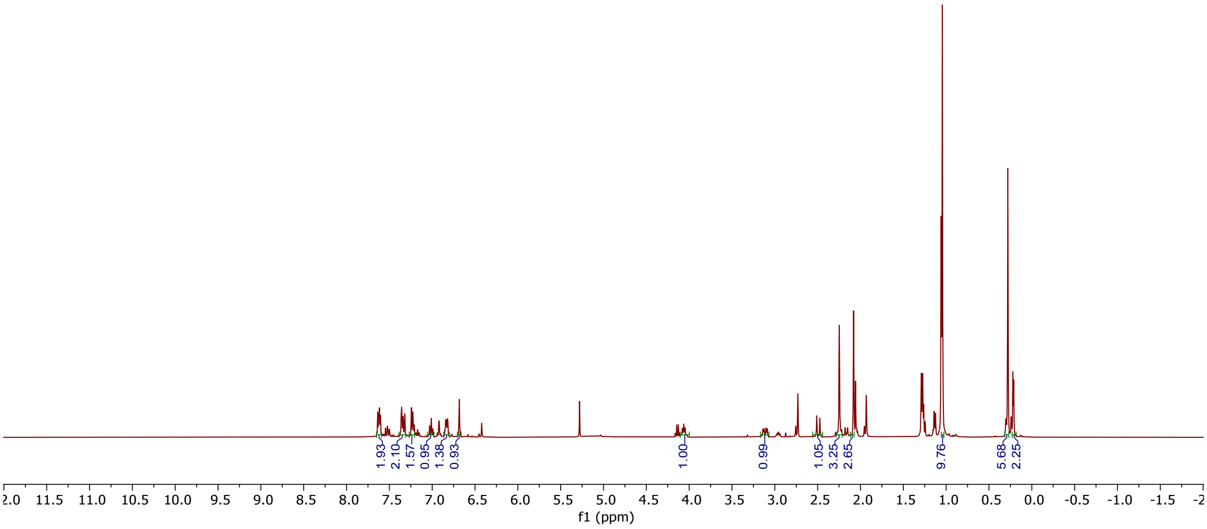


**Figure S6** $^{1}$H-NMR spectrum of **compound *E*-7** in $CDCl_3$.

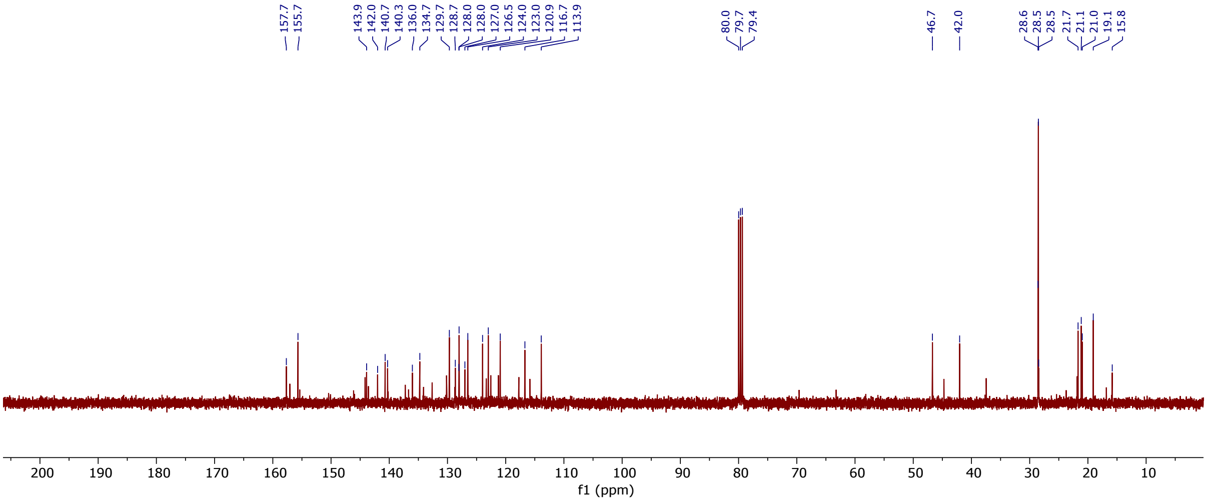


**Figure S7** $^{13}$C-NMR spectrum of **compound *E*-7** in $CDCl_3$.

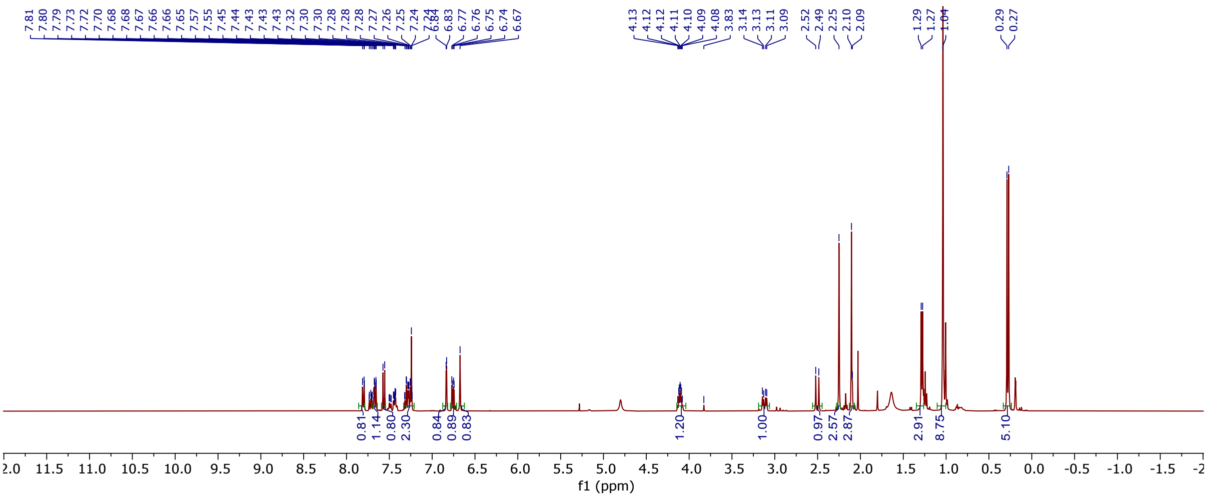


**Figure S8** $^{1}$H-NMR spectrum of **compound *Z*-7** in $CDCl_3$.

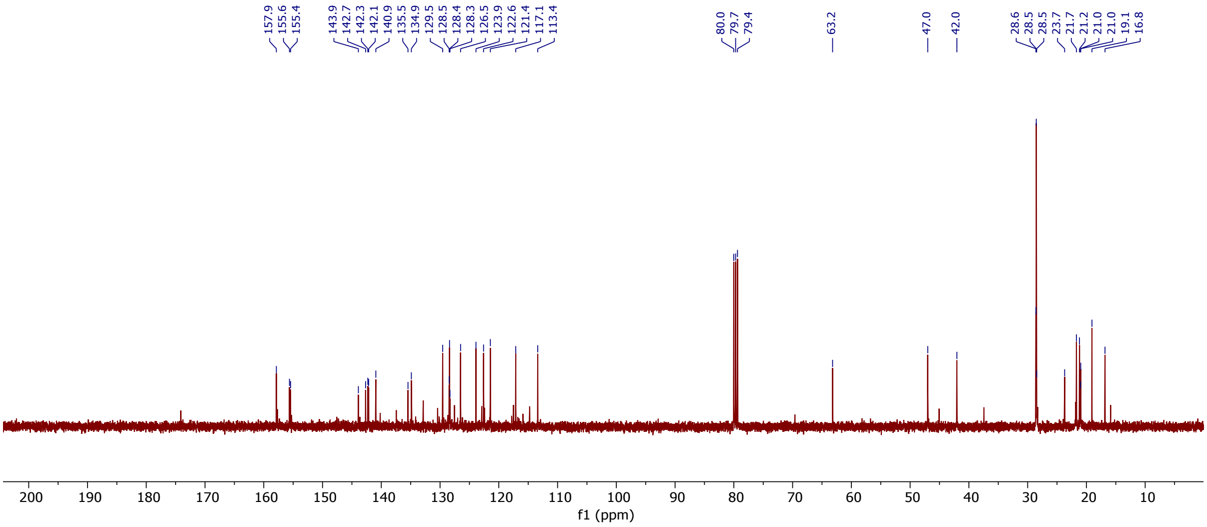


**Figure S9** $^{13}$C-NMR spectrum of **compound *Z*-7** in $CDCl_3$.

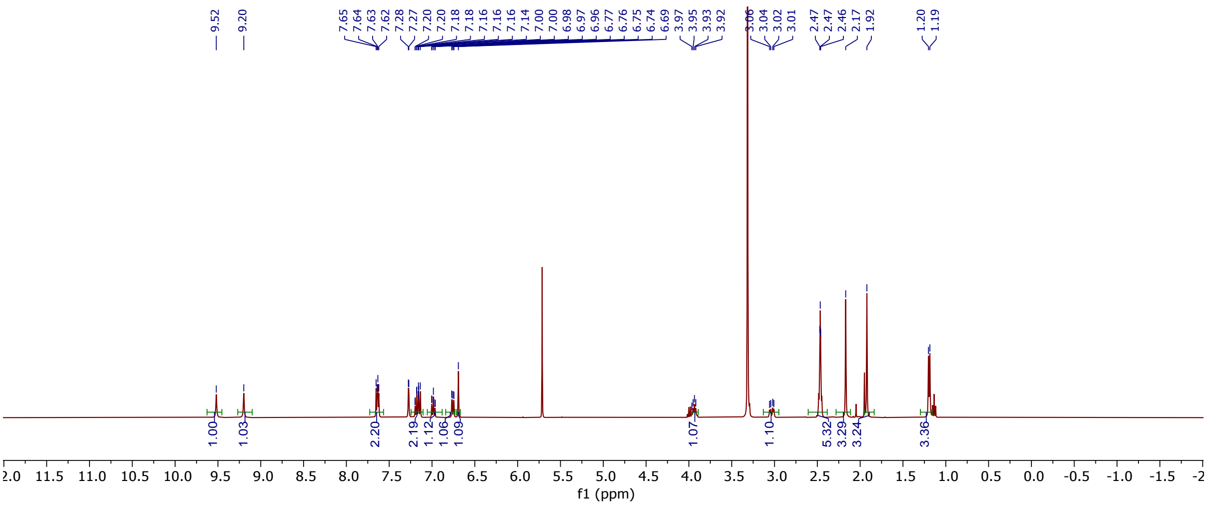


**Figure S10** $^{1}$H-NMR spectrum of **compound *E*-8** in DMSO-$d_6$.

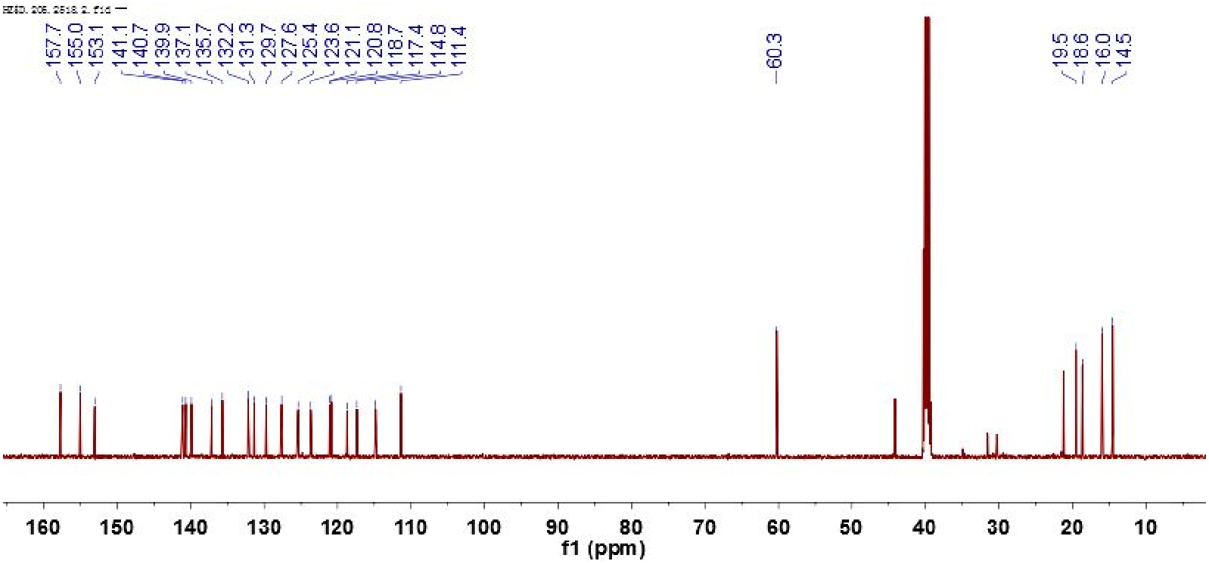


**Figure S11** $^{13}$C-NMR spectrum of **compound *E*-8** in DMSO-$d_6$.

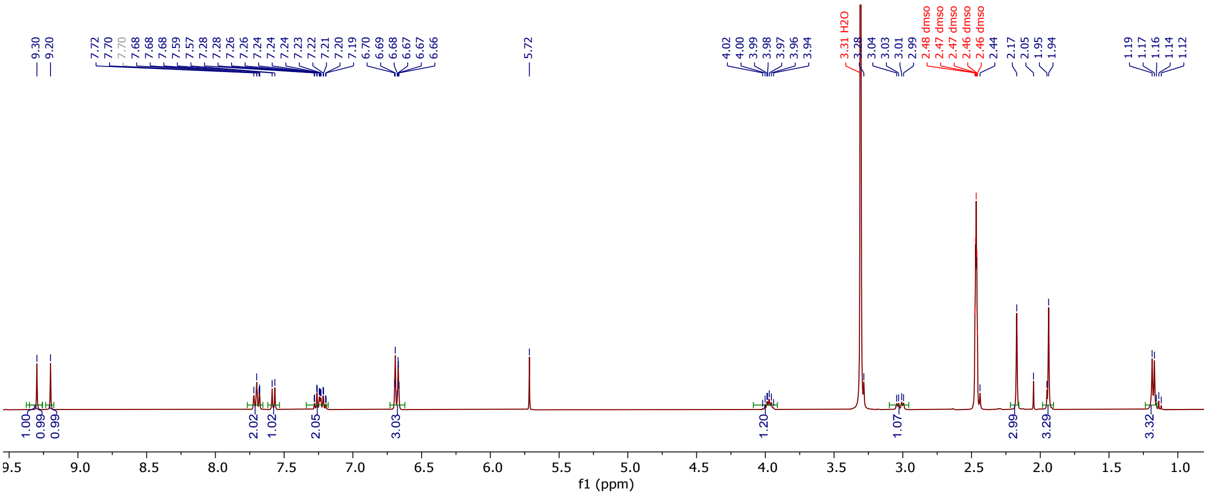


**Figure S12** $^{1}$H-NMR spectrum of **compound *Z*-8** in DMSO-$d_6$.

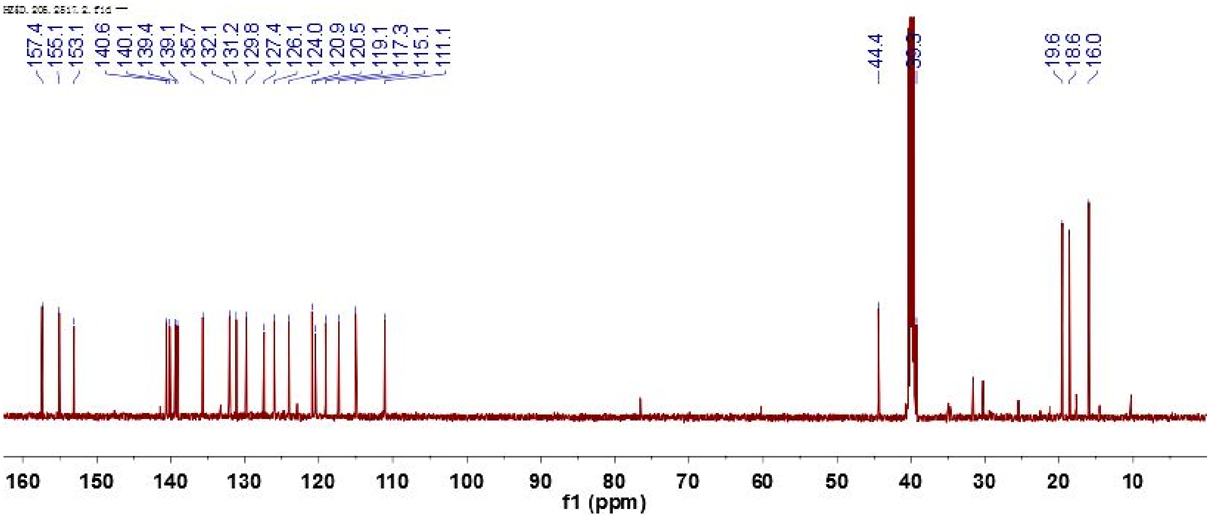


**Figure S13** $^{13}$C-NMR spectrum of ***compound Z*-8** in DMSO-$d_6$.

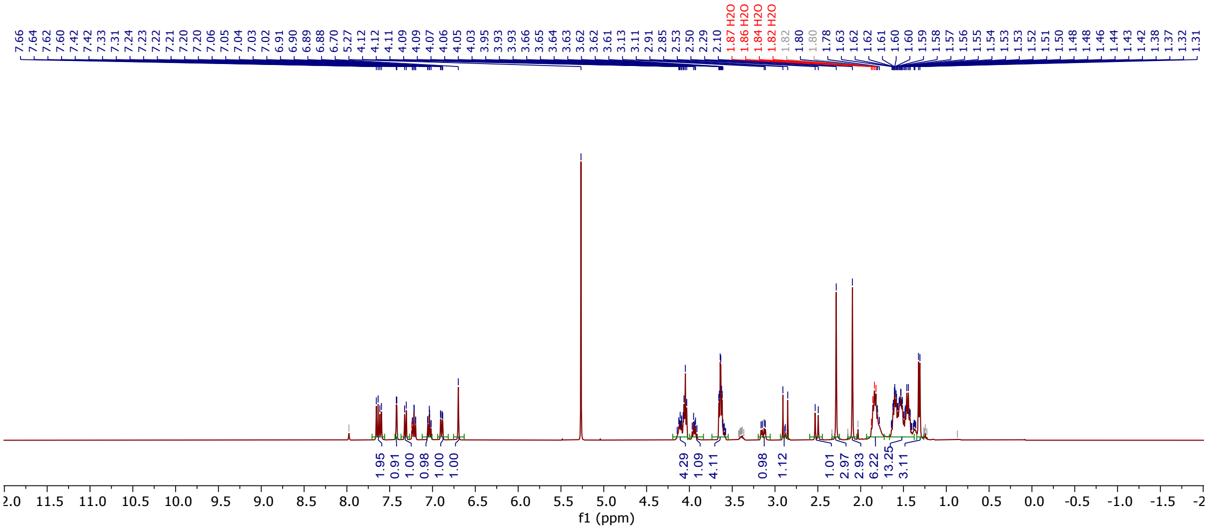


**Figure S14** $^{1}$H-NMR spectrum of ***compound E*-9** in $CDCl_3$.

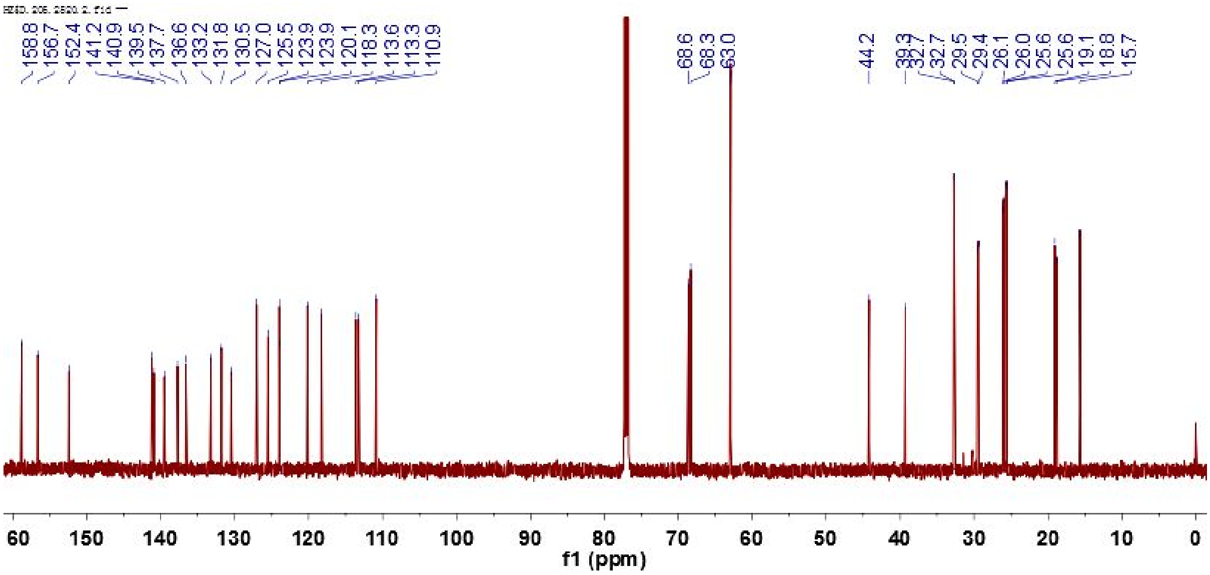


**Figure S15** $^{13}$C-NMR spectrum of ***compound E*-9** in $CDCl_3$.

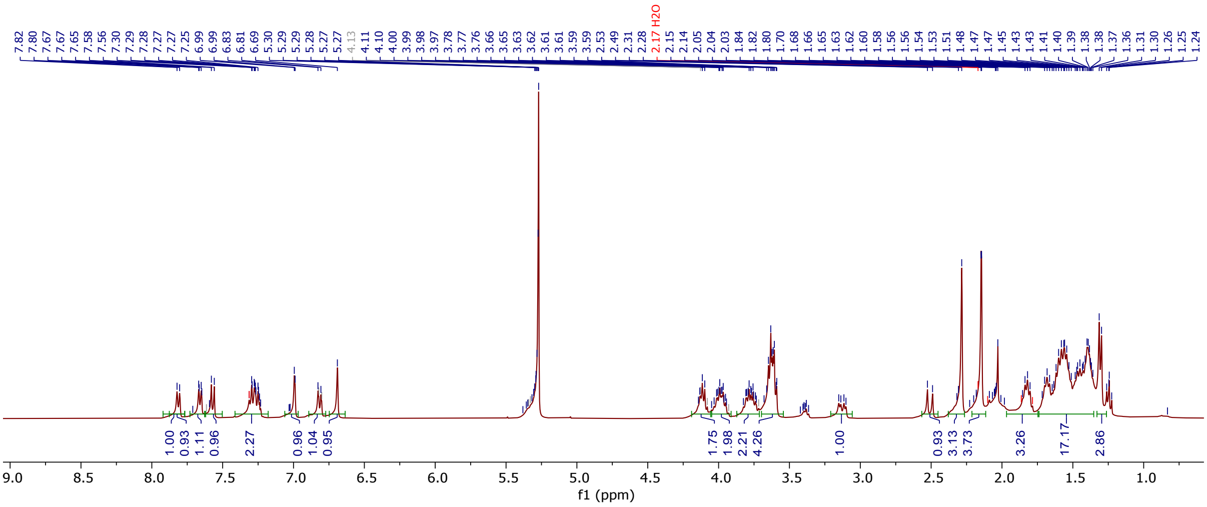


**Figure S16** $^{1}$H-NMR spectrum of **compound *Z*-9** in $CDCl_3$.

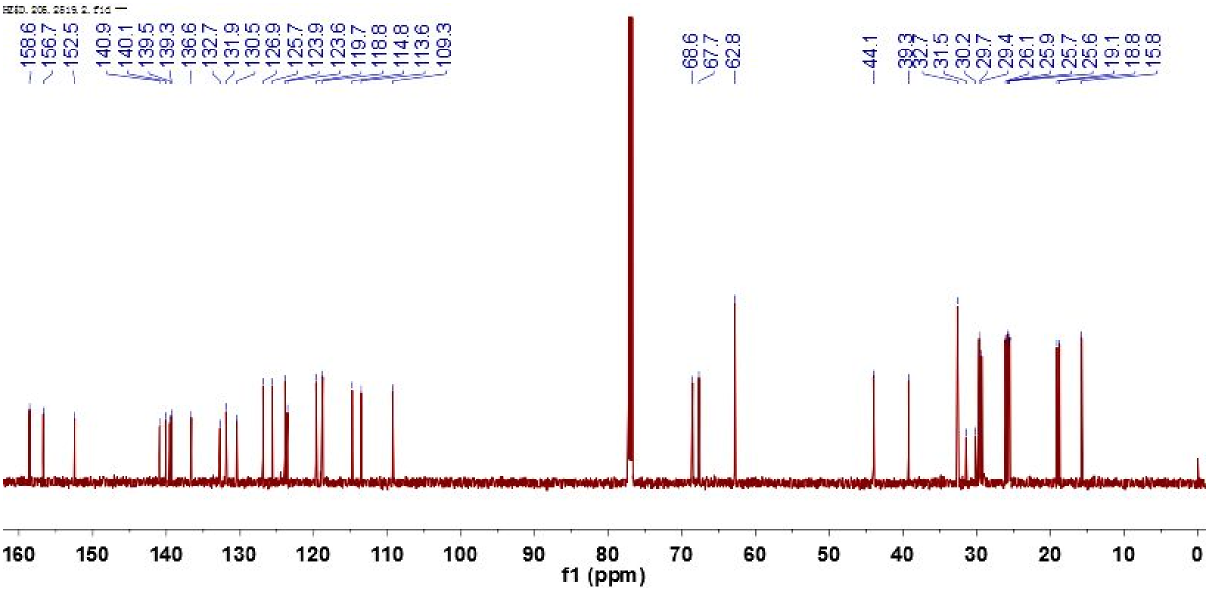


**Figure S17** $^{13}$C-NMR spectrum of **compound *Z*-9** in $CDCl_3$.

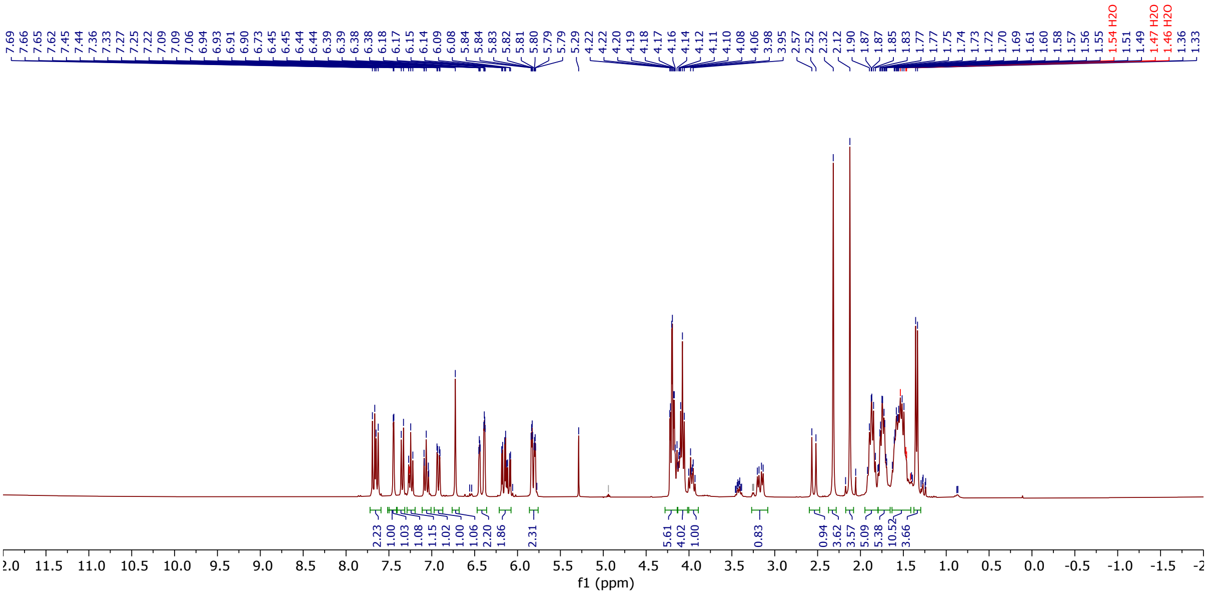

**Figure S18** $^{1}$H-NMR spectrum of ***compound E*-M1** in $CDCl_3$.

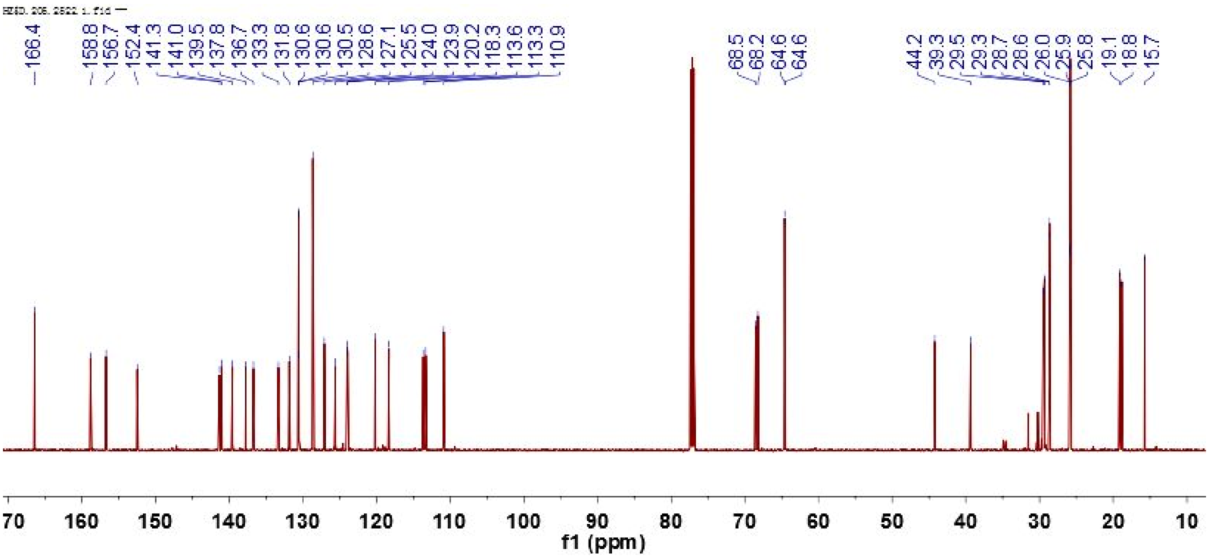


**Figure S19** $^{13}$C-NMR spectrum of ***compound E*-M1** in $CDCl_3$.

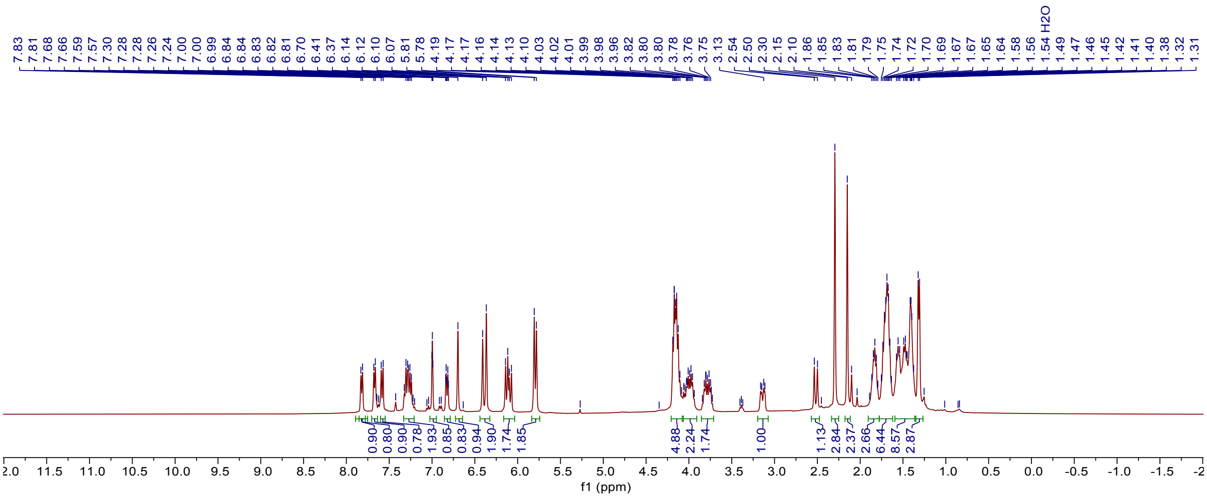


**Figure S20** $^{1}$H-NMR spectrum of ***compound Z*-M1** in $CDCl_3$.

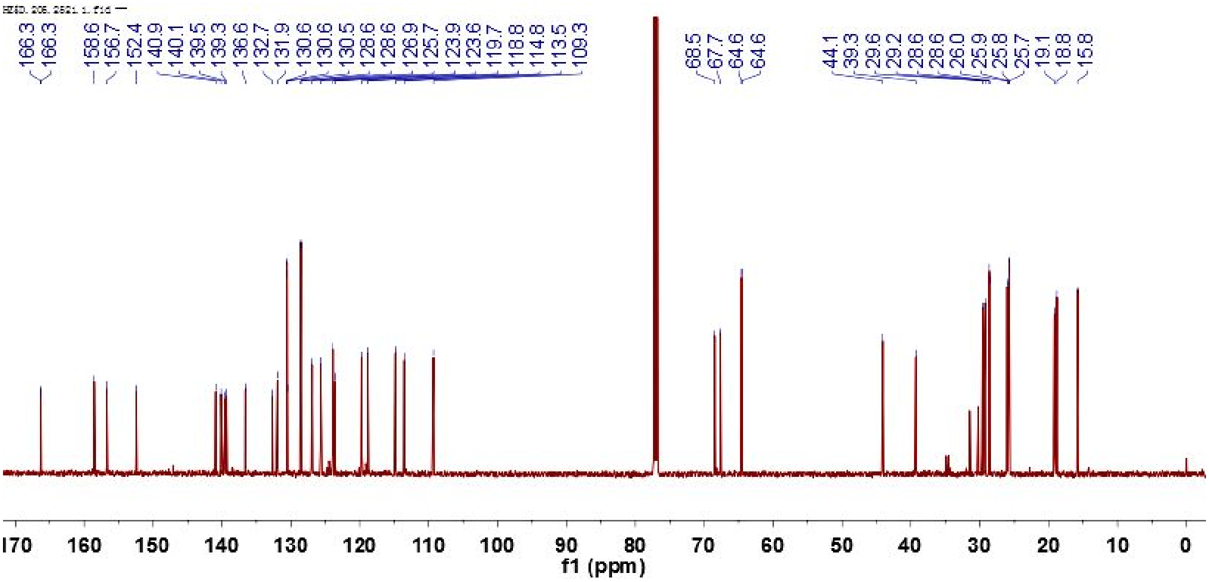

**Figure S21** $^{13}$C-NMR spectrum of **compound *Z*-M1** in $CDCl_3$.

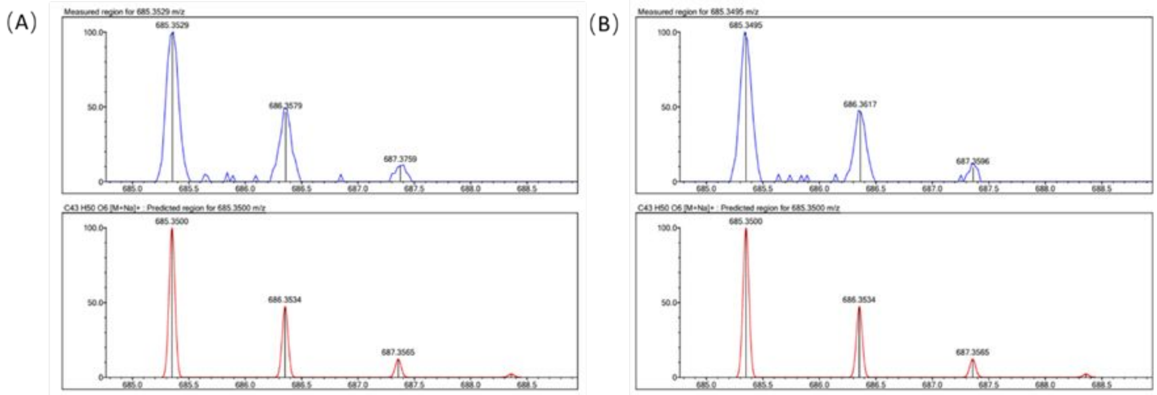

**Figure S22** HRMS spectrum of (A) compound ***E*-M1** and (B) compound ***Z*-M1**

## Photochemical and Thermal Behavior of Molecular Motor

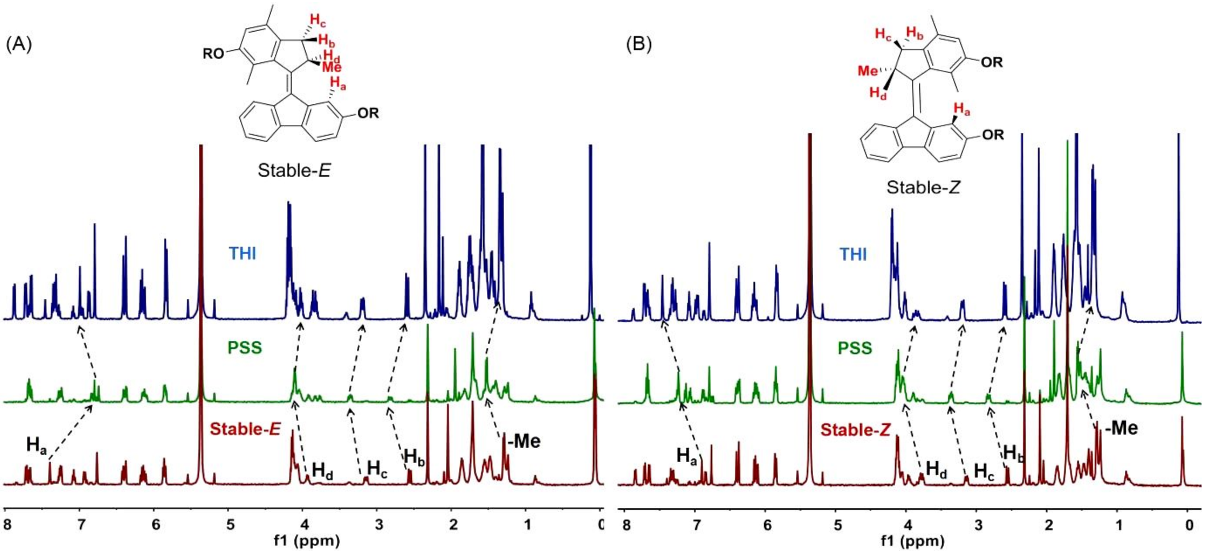


**Figure S23** Irradiation study of molecular motor in DCM-$d_2$. (A) ***E*-M1** (red) was irradiated with 365 nm light at -40 $^oC$ for 4 h until no further change was observed (green). Thermal helix inversion process was recorded by keeping sample at rt for 2 h (blue). (B) ***Z*-M1** (red) was irradiated with 365 nm light at -40 $^oC$ for 4 h until no further change was observed (green). Thermal helix inversion process was recorded by keeping sample at rt for 2 h (blue). Sample concertation was 2 ×10 $^{-3}$ M.

## UV-vis and CD study

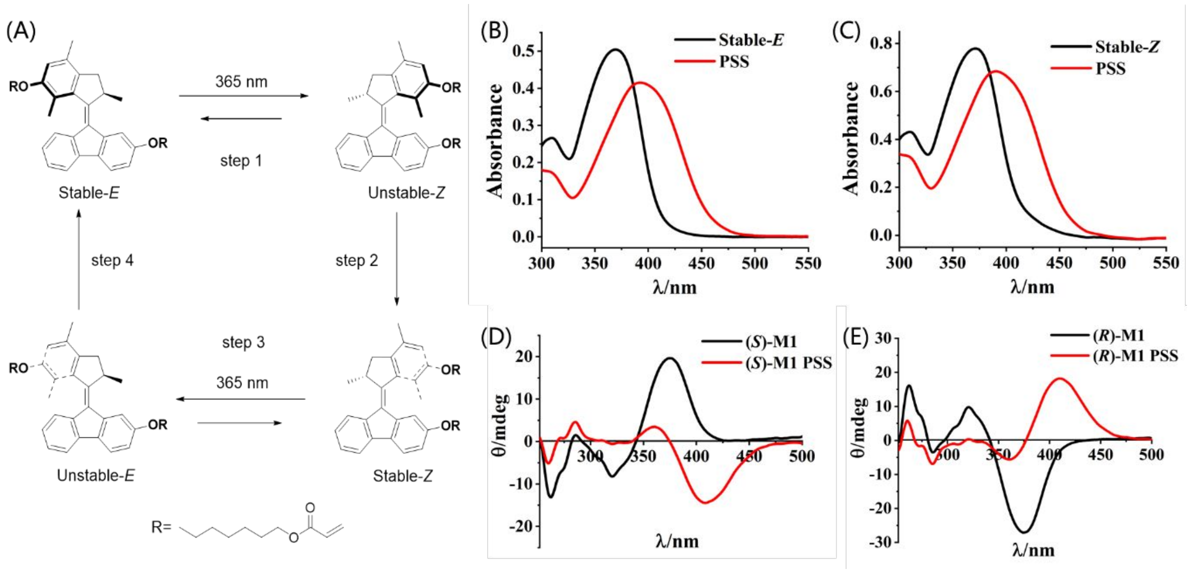


**Figure S24** (A) Full 360° rotary cycle for Molecular Motor **M1**. (B) UV-vis spectra of stable-*E* in DCM at 253 K upon UV irradiation ($3.8\times10^{-5}$ M). (C) UV-vis spectra of stable-*Z* in DCM at 253 K upon UV irradiation ($5\times10^{-5}$ M). (D) CD spectra of (*S*)-**M1** in DCM at 253 K upon UV irradiation ($3.8\times10^{-5}$ M). (E) CD spectra of (*R*)-**M1** in DCM at 253 K upon UV irradiation ($5\times10^{-5}$ M).

## Kinetic Study

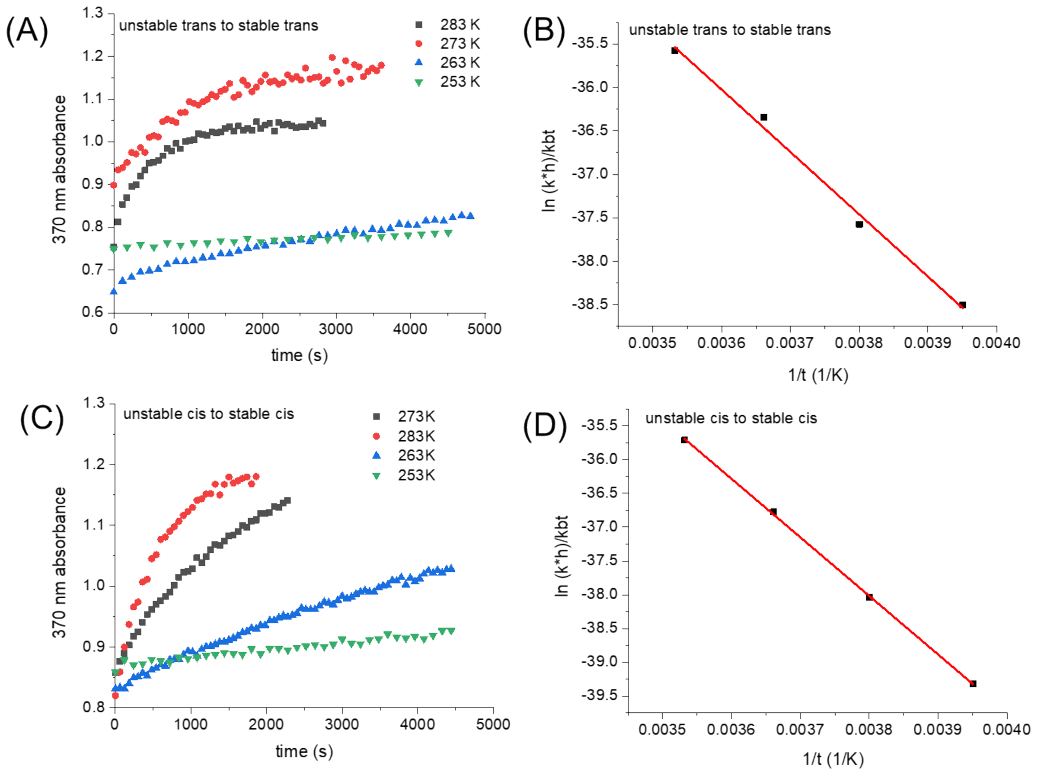


**Figure S25** Eyring plot for the thermal helix inversion of unstable-***E-M1*** to stable-***E-M1***. (A) The rate constants of the first-order decay *k* were obtained from equation $A/A_o= e^{-kt}$, at 283 K,

273 K, 263 K and 253 K. (B) The linear fitting of ln ($k$h/k$_0$T) by 1/T using Erying equation $\ln\frac{k}{T}=\frac{-\Delta H^{\ddagger}}{R}\cdot\frac{1}{T}+\ln\frac{k_B}{h}+\frac{\Delta S^{\ddagger}}{R}$. Eyring plot for the thermal helix inversion of unstable-***Z-M1*** to stable-***Z-M1***. (C) The rate constants of the first-order decay $k$ were obtained from equation $A/A_o= e^{-kt}$, at 283 K, 273 K, 263 K and 253 K. (D) The linear fitting of ln ($k$h/k$_0$T) by 1/T using Erying equation $\ln\frac{k}{T}=\frac{-\Delta H^{\ddagger}}{R}\cdot\frac{1}{T}+\ln\frac{k_B}{h}+\frac{\Delta S^{\ddagger}}{R}$.

**Preparation of liquid crystal polymer with racemic motors**

Two glass substrates were thoroughtly cleaned and spin-coated with polyvinyl alcoholsolution (5 wt% PVA in water). After removing all the water at 100 $^{o}$C, the substrates were rubbed at velvet to form the alignment layer. Two glass plates with the same alignment layer were sticked together perpendicularlly and fixed by the UV-curing spacer (18 μm) , to form a LC cell. Afterward, the LC mixture (3 wt% stable-*E* **M1** with 18 wt% RM 23, 31 wt% RM 82, 46 wt% RM 105 and 2 wt% IRG 819) was filled into the cell at 80 $^{o}$C by capillary suction. The cells were subsequently cooled down to 40 $^{o}$C. During this process, the filled liquid crystal mixture undergoes a change from an isotropic phase to a nematic phase at around 60 $^{o}$C (Figure S26) and the cell configuration enables the LC mixture containing motors to align in a twisted nematic manner. Next the mixture was copolymerized using blue (455 nm) light irradiation with a light intensity of 80 mw/cm$^{2}$. The liquid crystal films were annealed at 125 $^{o}$C for 10 min and cooled down to rt. The DSC curve shows that the glass transition temperature ($T_g$) of the polymeric film is around 27 $^{o}$C (Figure S27).

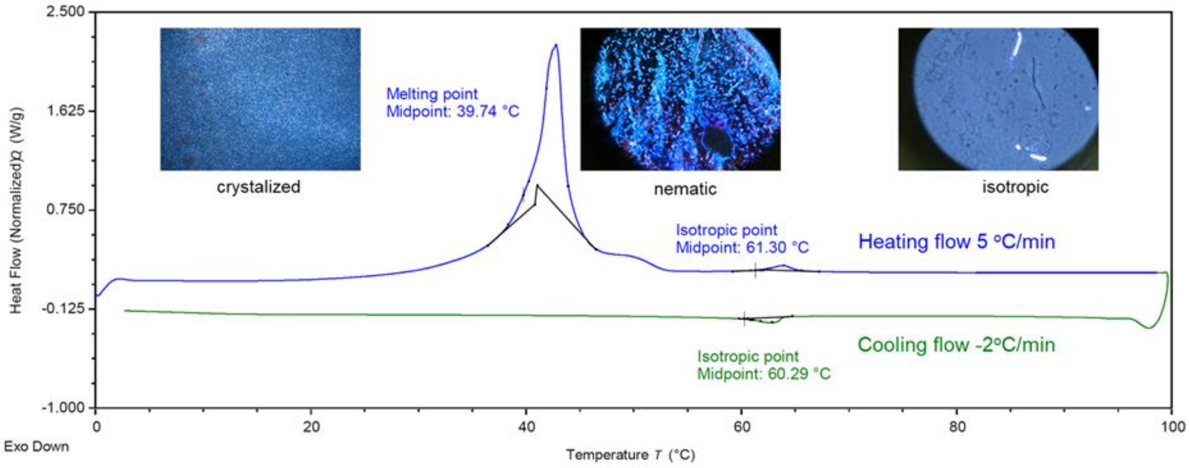


**Figure S26** DSC curve of liquid crystal mixture. The sample was heated from 0 to 100 $^{o}$C with a heating rate of 5 $^{o}$C and then cooled down to 0 $^{o}$C with a cooling rate of -2 $^{o}$C.

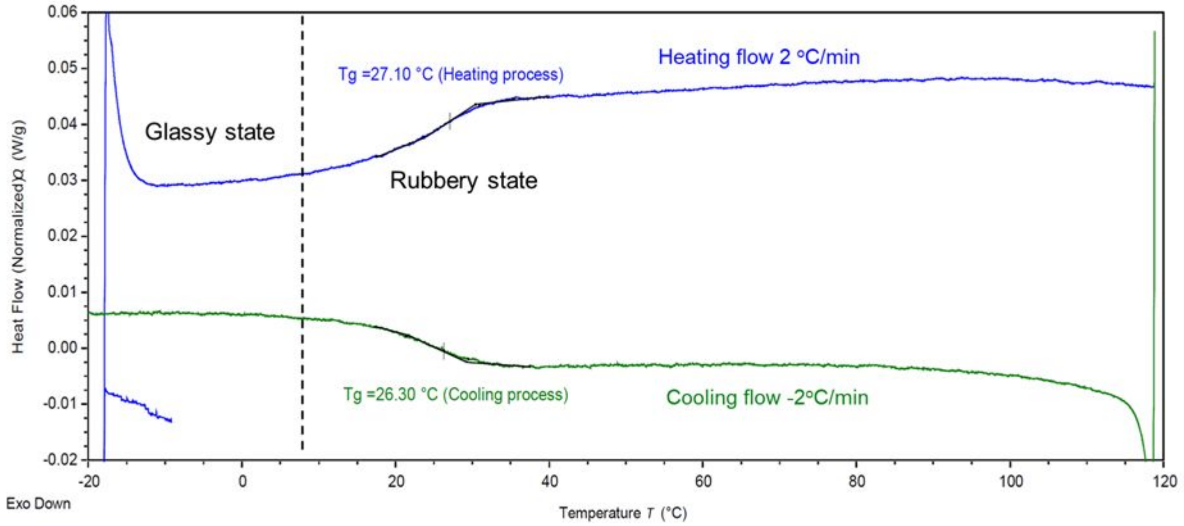


**Figure S27** DSC curve of polymeric film. The sample was heated from -20 to 120 °C with a heating rate of 2 °C and then cooled down to -20 °C with a cooling rate of -2 °C.

**UV-Vis study of LC polymeric film**

Upon UV light (365 nm) illumination, the UV-vis absorption of the film shows a decrease at 380 nm with a concomitant increase at 430 nm (Figure S28 A), which is similar to the change of the motor in solution. It indicates the rotary motion of the motor in the LC ribbon during the irradiation. When the UV light was switched off, the original spectra were regained. The photo-actuation and recovery have been repeated several times and the system operates without any fatigue during the cycles (Figure S28 B).

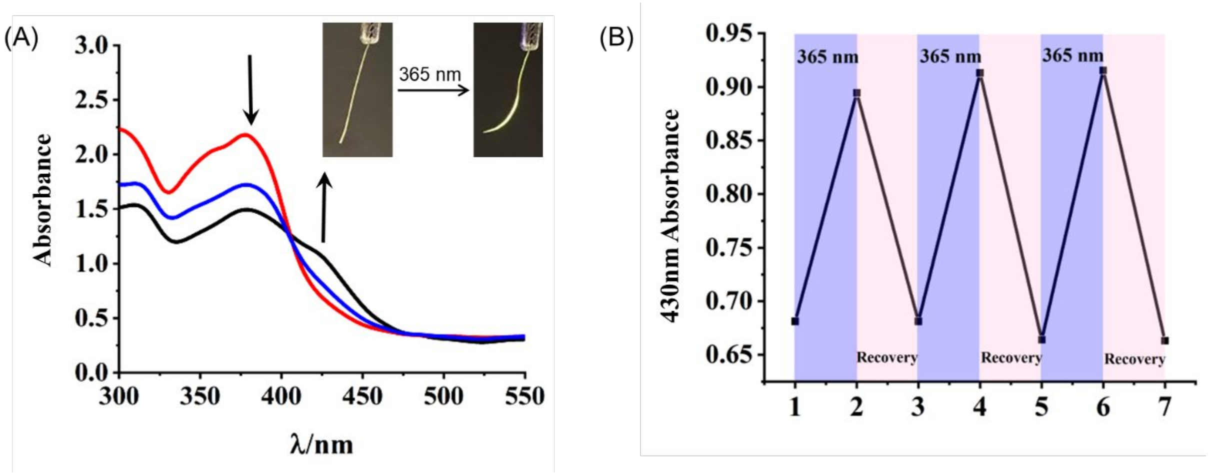


**Figure S28** (A) UV-vis spectrum of polymeric film before and upon light irradiation at rt. Film sample was fixed with a spacer and was irradiated until no further change observed. (B) UV-vis absorptions of the LC ribbon at 380 nm during the irradiation cycles. The ribbon is irradiated with the UV light (365 nm) ON and OFF for 3 cycles.

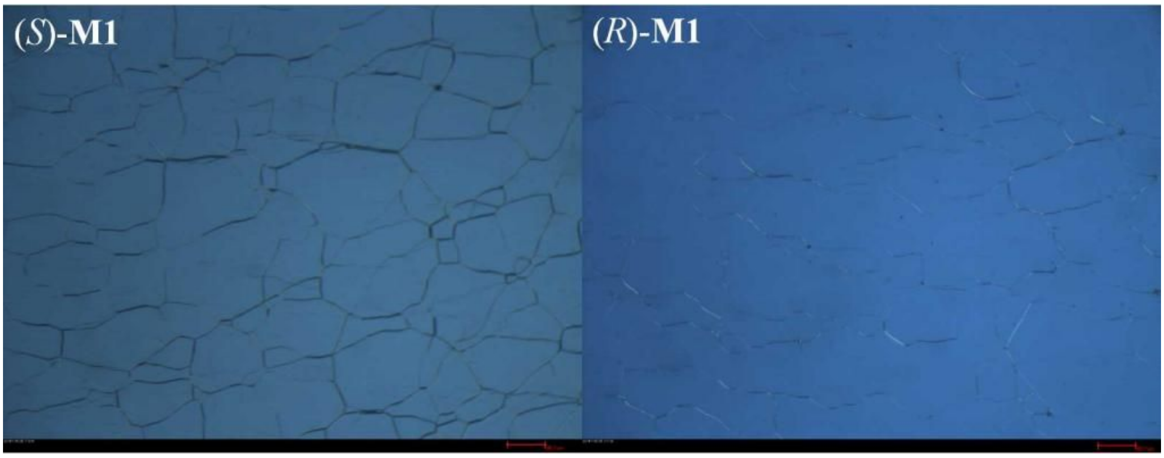


**Figure S29** POM image of the LC film. The chiral doped LC material was prepared from 3 wt% of (*S*)-**M1** (left) or 3 wt% of (*R*)-**M1** (right) with the LC monomer mixture (18 wt% RM 23, 31 wt% RM 82, 46 wt% RM 105 and 2 wt% IRG 819). The resulted LC mixture was filled in a 20 μm planar cell.

**Preparation of liquid crystal polymer film containing chiral motor with complex orientation**

**LC film with "cross" shape**

The glass substrate with SD1 is exposed to 405 nm ultraviolet linearly polarized light under a photomask. After the first exposure, the linearly polarized light and the mask were rotated 90° clockwise, and then the glass substrate were exposed for the second time (Figure 4C), each exposure time was 60 s. Then two resulting glass substrates were glued together with a fixed thickness of 50 μm to afford a cell with planar alignment in different directions. A mixture of 3 wt% motor M1 (which contains 1 wt% optically pure motor), 18 wt% RM 23, 31 wt% RM 82, 46 wt% RM 105 and 2 wt% IRG 819 was filled into a cell. Samples were exposed to UV at 40 $^oC$ for 5 min with the mercury lamp at light intensities 200 mW/cm$^2$. The lamp was equipped with a cut-off filter and it can only transmit light with a wavelength of 455 nm. After the polymeric films were formed, they were cut along the "designed" directions of alignment layers to obtain the ribbons with "cross" shapes.

**LC film with "flower" shape**

The glass substrate with SD1 is exposed to 405 nm ultraviolet polarized light under a photo mask. After the first exposure, the linearly polarized light and the mask were rotated clockwise by 45°, 90°, and 135°, and the glass substrate is sequentially exposed, and the time for each exposure is 60 s. The resulting two glass substrates were glued together with a fixed thickness of 50 μm to produce a cell with planar arrangements in different directions. Similarly, the liquid crystal mixture was filled into cells. After the sample was cured by UV light, a liquid crystal polymer film was formed, which was cut along the "designed" directions of the alignment layer, and the "flower" shaped strips can be obtained.

**Control experiments**

1. The potential heating was monitored during light irradiation using an infrared camera.

The liquid crystal polymer film (50 μm) embed with motors was placed in the air and irradiated with UV light of the same intensity as used for **M1** while the film was monitored in real time with an infrared camera. It was found that during the process of actuation, the temperature of the film was kept at rt. Therefore, the deformation of the film is due to the rotary motion of molecular motor, rather than the thermal effect.

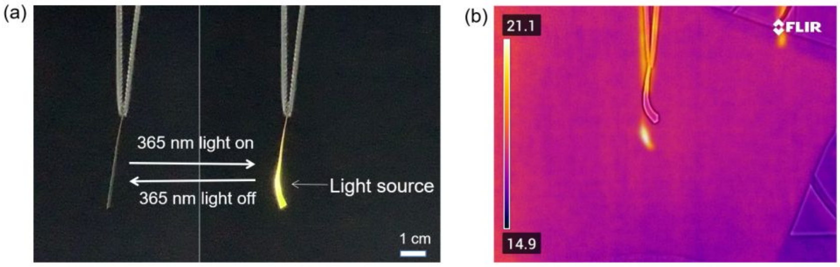


**Figure S30.** Heating image of LC ribbon by infrared camera after irradiation.

2. Studying the actuation in submerged conditions.

The ribbon was brought into water and actuation was observed after UV irradiation. It indicates the actuation is driven by rotary motion of molecular motor inside and not by the photothermal effect.

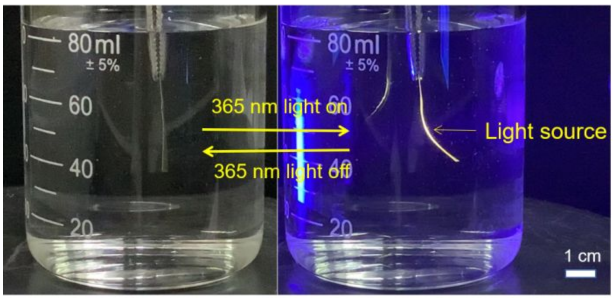


**Figure S31.** LC ribbon based on molecular motors before and after UV irradiation at rt under water.

3. Control compound with only one side acrylate group

We have prepared the motor that contains the same core but with only one acrylate group for polymerization. The control film was formed under identical conditions as those of **M1** and studied under the same photochemical conditions. However, no shape change was observed after UV light irradiation using this control compound not able to function as a cross-linking unit in a liquid crystal network. It strongly supports the notion that the observed actuation of the LCN ribbon based on motor **M1** is predominantly due to the rotation and change in shape of the motor, and its effect on the order parameter of the LCN and cannot be attributed to a heating effect.

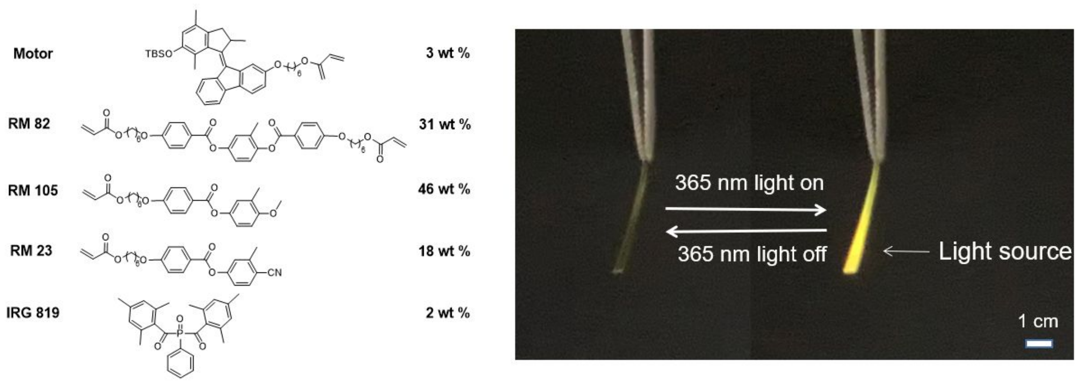


**Figure S32.** UV irradiation of LC ribbon embedded with motor that contains one side acrylate group.

**Reference:**

[1] Neubauer, T. M.; van Leeuwen, T.; Zhao, D.; Lubbe, A. S.; Kistemaker, J. C. M.; Feringa, B. L. Asymmetric synthesis of first generation molecular motors. *Org. Lett.* **2014,** *16* (16), 4220–4223.

[2] Hou, J.; Mondal, A.; Long, G.; de Haan, L.; Zhao, W.; Zhou, G.; Liu, D.; Broer, D. J.; Chen, J.; Feringa, B. L. Photo-responsive helical motion by light-driven molecular motors in a liquid-crystal network. *Angew. Chem. Int. Ed.* **2021,** *60* (15), 8251–8257.
